# Bikeability and the induced demand for cycling

**DOI:** 10.1073/pnas.2220515120

**Published:** 2023-04-11

**Authors:** Mogens Fosgerau, Mirosława Łukawska, Mads Paulsen, Thomas Kjær Rasmussen

**Affiliations:** ^a^Centre for Computational Economics, Department of Economics, University of Copenhagen, 1353 Copenhagen K, Denmark; ^b^Department of Technology, Management and Economics, Technical University of Denmark, 2800 Kgs. Lyngby, Denmark

**Keywords:** bicycles, travel demand, networks, infrastructure

## Abstract

To what extent is the volume of urban bicycle traffic affected by the provision of bicycle infrastructure? In this study, we exploit a large dataset of GPS trajectories of bicycle trips in combination with a fine-grained representation of the Copenhagen bicycle-relevant network. We apply a model for bicyclists’ choice of route from origin to destination that takes the complete network into account. This enables us to determine bicyclists’ preferences for a range of infrastructure and land-use types. We use the estimated preferences to compute a generalized cost of bicycle travel, which we correlate with the number of bicycle trips across a large number of origin–destination pairs. Simulations suggest that the extensive Copenhagen bicycle lane network has caused the number of bicycle trips and the bicycle kilometers traveled to increase by 60% and 90%, respectively, compared with a counterfactual without the bicycle lane network. This translates into an annual benefit of €0.4M per km of bicycle lane owing to changes in generalized travel cost, health, and accidents. Our results thus strongly support the provision of bicycle infrastructure.

## Significance

Promotion of bicycle use has received considerable global attention. In addition to reducing the impact of urban transportation on climate, it will help to improve public health and reduce traffic congestion, noise, and air pollution. Provision of bicycle-friendly infrastructure is a primary means to achieving this. Using a large dataset of GPS trajectories of bicycle trips and fine-grained network data covering the city of Copenhagen, Denmark, this study finds a large effect of infrastructure provision on the volume of bicycle traffic.

Provision of bicycle infrastructure is a means to encourage cycling, which may in turn improve the urban environment and reduce the impact of transport on climate (1). Even though bicycle infrastructure is relatively cheap, it does come at a cost and takes up valuable urban space. Therefore, assessing the impact of providing bicycle infrastructure on cycling is of interest, taking into account the importance of location and type of infrastructure (2).

**Fig. 1.**
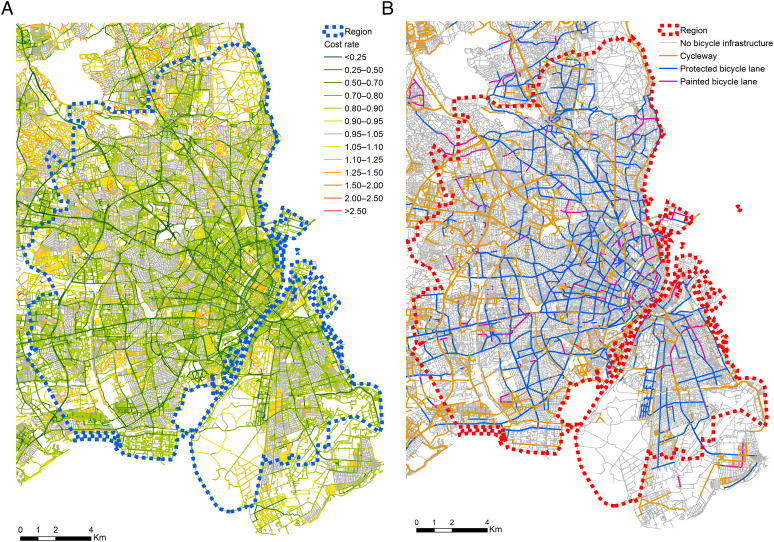
Maps representing the network used in the case study.

Copenhagen has extensive bicycle infrastructure and a high level of bicycle usage for everyday urban travel (3). We have a huge dataset comprising the GPS trajectories (also known as trip chains) of 218,489 bicycle trips (point-to-point movements) in Copenhagen obtained from users of Hövding airbag helmets (4). Matching these trajectories with very detailed network information (Fig. 1*A*) allows us to track the observed bicycle route choices across a range of infrastructure and land use types.

To make inference regarding the factors influencing bicyclists’ choice of routes, we must compare the observed chosen routes to the possible alternatives. However, the number of possible routes between two points in a large network is extremely large and impossible to enumerate. To overcome this, we exploit the recently proposed perturbed utility route choice model (5), which allows the entire network to be taken into account while being computationally feasible.

The estimated route choice model indicates very substantial variation in the generalized cost, which weighs the preferences for different infrastructure types. The generalized cost per meter traveled on the most attractive infrastructure type, a cycleway, designated as a cycle superhighway and located in a green area, is seven times lower than the generalized cost of cycling on a residential road.

Bicycle infrastructure can therefore considerably affect the generalized cost of cycling and thereby the volume of bicycle use. We relate the number of trips in each origin–destination (OD) pair to the generalized cost from the route choice model. Employing a variant of the gravity model (6), we find a clear relationship whereby lower generalized cost is associated with a higher number of bicycle trips. We use this model to simulate a range of counterfactual scenarios, exploring the impact of the bicycle network on the volume of bicycle use.

## Results

### Bicycle Route Choice.

We employ the perturbed utility route choice model (5). The network links are indexed by *e* ∈ ℰ. For each OD pair, the model predicts a flow vector $x={\left(x_{e}\right)}_{e\in E}\in R_{+}^{\left|E\right|}$ that is physically consistent with a flow of mass one through the network from origin to destination. The link flow *x*_*e*_ is the share of the flow that uses link *e*.

The model assumes that the flow minimizes a cost function

[1] $$\begin{array}{c} C\left(x\right)=\sum_{e\in E}l_{e}\left(c_{e}x_{e}+F\left(x_{e}\right)\right). \end{array}$$

We will explain the terms of the cost function in some detail.

To each link *e*, we associate a link cost rate *c*_*e*_ = *z*_*e*_′*β*, where *z*_*e*_ is a vector of link characteristics and *β* is a vector of the parameters to be estimated. The link cost rate does not involve monetary elements but is a generalized cost that represents the bicyclists’ preferences for various infrastructure types. Multiplying the link cost rate by the link length *l*_*e*_ and summing across links, we obtain the first component of the cost ∑_*e* ∈ ℰ_*l*_*e*_*c*_*e*_*x*_*e*_.

The first cost component is linear in the flow *x*, which means that the flow minimizing just that would concentrate on a single route. In reality, observed flows are distributed on several routes. This motivates the second term in the cost function, which is given in terms of a perturbation function defined by *F*(⋅):ℝ_+_ → ℝ by *F*(*x*_*e*_)=(1 + *x*_*e*_)ln(1 + *x*_*e*_) − *x*_*e*_. The function *F* is convex and increasing, which makes it costly to concentrate flow on few links and thereby induces a tendency for flow to distribute on more routes. On the other hand, *F*(0)=*F*′(0)=0, which allows the cost-minimizing flow to be zero on most links.

Estimation of the route choice model is based on the observed share of travelers in an OD pair that uses each link. The materials and methods section explains the data and estimation methods, and further details are provided in *SI Appendix*. The full specification of the vector of link characteristics *z*_*e*_ and the corresponding parameter estimates are given in *SI Appendix*, Table S3.

Fig. 1*B* shows the estimated link cost rates on a map of the network. The main bicycle network is clearly visible and seems to be quite dense and well connected. Such maps may be used by urban planners to suggest areas where the bicycle network could be improved. The specification of the cost rate comprises variables that indicate the type of infrastructure for each link in the network, including information about the type of bicycle infrastructure, and the nearby land use. We discuss the most important parameters in turn.

The reference category is residential roads without bicycle infrastructure in low-rise urban areas. Compared with the reference, bicyclists associate a 11% higher cost rate with large roads (roads with at least two lanes in one direction), whereas the difference to medium roads (roads with at most one lane in each direction) is small and statistically insignificant. We do not have data for the volume of car traffic, so we must rely on the road type as proxy. These results thus suggest that bicyclists associate a higher generalized cost to roads with more car traffic.

Provision of dedicated bicycle infrastructure quite substantially reduces the generalized cost of bicycling. Cycleways (bicycle paths in own trace) reduce the generalized cost by 20%. On residential and medium roads, bicycle lanes, whether protected or just painted, reduce the cost rate by 14% and 22%, respectively. The type of bicycle lane has a considerable effect on the cost rate for the large roads category: Painted bicycle lanes have only a small and statistically insignificant effect, whereas protected bicycle lanes reduce the cost rate by 34%. It makes clear intuitive sense that the impact of bicycle lanes is larger, the larger the road is. In particular, only protected lanes affect the generalized cost on largest roads where car traffic is heavier.

A number of routes are branded as so-called cycle superhighways. This is a label given to high-quality, continuous bicycle routes, that cater to commuter cyclists (7). Additional routes are planned to become cycle superhighways in the future but have not yet received the label (8). We estimate a cost rate reduction of 12%, both for the actual and the planned cycle superhighway links. This suggests that the routes included in the cycle superhighway network were ex ante attractive and that the transformation from planned to actual cycle superhighway does not yield any additional cost reductions beyond those already accounted for at the link level.

The model also includes parameters accounting for interactions between the type of infrastructure and the neighboring land use. The cost rate is much reduced for cycleways in industrial areas (48%) or green areas (53%). It makes intuitive sense that cycleways in green areas may be pleasant. Another potential explanation which also applies to industrial areas is the attractiveness of isolation from heavy traffic.

In summary, provision of bicycle-friendly infrastructure has a substantial effect on route choice. We shall see below that this translates into a substantial effect on the number of bicycle trips.

### Bicycle Travel Demand.

We set up a gravity model to measure the association between the number of bicycle trips in each OD pair and the characteristics of the bicycle network. Where the route choice model is based on the observed share of travelers in an OD pair that uses each link, the demand model, in contrast, is based on the number of travelers in each OD pair. The route choice model parameter estimates show that the characteristics of the network significantly affect the generalized cost of using the links of the network. Therefore, the route choice model can be used to compute the generalized cost of traveling by bicycle in any given OD pair, thereby aggregating the network information in a model-consistent manner.

Let ${\hat{c}}^{\mathrm{od}}=\sum_{e\in E}l_{e}\left(c_{e}{\hat{x}}_{e}^{\mathrm{od}}+F\left({\hat{x}}_{e}^{\mathrm{od}}\right)\right)$ be the generalized cost associated with the cost-minimizing flow ${\hat{x}}^{\mathrm{od}}$ connecting origin *o* to destination *d* and let *Y*^*od*^ be the observed number of trips in the OD relation *od*. We assume that *Y*^*od*^ follows a Poisson distribution with expectation given as a log-linear function of the cost:

[2] $$\begin{array}{c} \ln E\left[Y^{\mathrm{od}}\right]=D\left({\hat{c}}^{\mathrm{od}}\right)+\delta +\eta_{o}+\gamma_{d}, \end{array}$$

where *δ* is a constant, and *η*_*o*_ and *γ*_*d*_ are constants for each origin and destination, respectively, except one. The constants account for the total bicycle traffic volume out of each origin and the total volume into each destination. The demand function *D* is expected to be downward sloping, such that higher cost implies less volume. We specify *D* to be continuous and piecewise linear with the number of pieces chosen by eye-balling.

**Fig. 2.**
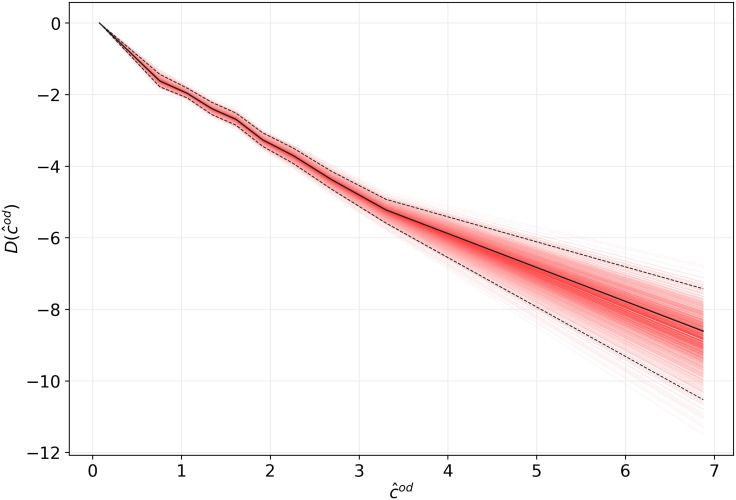
Estimate of the demand function *D* as a function of the generalized cost ${\hat{c}}^{\mathrm{od}}$ for OD pair *od*. The piecewise linear specification of *D* uses 10 kn (*n* = 38, ​649, *R*^2^ = 0.43, and RMSE (in levels)=10.6). The red lines trace pointwise confidence intervals at various quantiles computed using 1,000 bootstraps. The black dashed lines indicate the estimated 95% pointwise confidence band.

Fig. 2 shows the estimated demand function *D* from Eq. **2**. As expected, the demand decreases monotonously as the generalized cost increases. This implies for each OD that the expected number of bicycle trips increases if the generalized cost of bicycling is reduced, e.g., through the provision of bicycle infrastructure. The figure also shows a bootstrapped pointwise confidence band for the estimate of the demand function. The confidence band is very tight at small values of ${\hat{c}}^{\mathrm{od}}$ and is wider at larger values where observations are fewer. From the estimated demand function, we can compute the implied demand elasticity as a function of the cost. We find that the demand elasticity decreases almost linearly from 0 to about −6.5 as the cost increases from 0 to 7. A 10% increase in the cost of a short trip thus has very little impact on the number of bicycle trips while it reduces the number of trips by up to 65% for the longest trips.

### Counterfactuals.

We now exploit our model to simulate the impact of general counterfactual changes to the bicycle-relevant network to illustrate how much bicycle infrastructure has contributed to encouraging bicycling in Copenhagen. These results may be of interest for other cities aiming to improve or expand their bicycle-relevant network.

Table 1 summarizes the results for the counterfactual scenarios. We compute the change in consumer surplus for bicyclists as well as the change in external cost owing to health and accidents. We have scaled the gravity model output such that the base scenario reflects the total number of kilometers traveled by bicycle in the region. A full economic evaluation of constructing bicycle infrastructure would also need to take into account construction costs, the effects on travel times by car, and the induced effects on climate, accidents, noise, and air pollution.

**Table 1.** Key figures of three counterfactuals

Scenario	No bicycle lanes	Painted lanes instead of protected lanes on large roads	No existing and planned cycle superhighway classifications
Length of network changed [km]	1,427.8	407.0	*333.7
Avg. bicycle travel cost [rel. increase, %]	34.3	7.1	12.8
# trips [relative decrease, %]	37.0 [*35.1, 38.6*]	9.9 [*7.2, 12.1*]	16.8 [*14.3, 18.8*]
Total distance traveled by bicycle [relative decrease, %]	46.8 [*44.9, 48.7*]	13.7 [*10.9, 16.2*]	22.8 [*20.2, 25.2*]
Loss of consumer surplus [M € / year]	174.1 [*171.8, 176.2*]	44.8 [*44.1, 45.5*]	75.6 [*74.6, 76.6*]
Loss owing to health and accidents [M € / year]	435.3 [*418.1, 452.9*]	127.8 [*101.1, 150.9*]	212.5 [*188.3, 234.4*]
Societal loss [M € / year]	609.4 [*594.3, 624.9*]	172.6 [*146.7, 195.0*]	288.1 [*265.0, 309.0*]
Loss relative to length of network changed [k € / year / km]	426.8 [*416.2, 437.7*]	424.0 [*360.5, 479.0*]	*863.6 [*794.1, 926.0*]

Numbers in square brackets indicate 95% bootstrap confidence intervals based on 1,000 full-sample repetitions. ^*^Measured as route distance rather than network distance.

The first counterfactual simulates a situation where all 1,427.8 km of bicycle lanes and all cycle superhighway classifications have been removed. On average, this increases the generalized cost of bicycling by 34.3% per km, which induces a decrease of 37.0% in the number of bicycle trips and 46.8% in the total distance traveled by bicycle. The bootstrapped confidence intervals indicate that these numbers are quite precisely determined. Relative to the situation without bicycle lanes, the simulation thus suggests that the provision of bicycle lanes has induced an increase in bicycle use by 59% (trips) and 88% (total distance traveled).

To measure the loss to bicyclists in the counterfactual scenario compared with the base scenario, we have computed the change in the consumer surplus (9). To convert this number from generalized cost units to monetary values, we first apply the sample average speed to convert the generalized cost to time units and then apply the official Danish value of travel time (10) to convert from time units to monetary units. Our results suggest a decrease in consumer surplus of €174.1M per year.

Bicycling is associated with both health benefits and accident risk, e.g., refs. 11 and 12. The official Danish guidelines for cost–benefit analysis suggest a net external benefit owing to health and accidents of 0.91 EUR per bicycle km (10). Applying this figure, we estimate the welfare loss induced by removing the bicycle lane network through health and accidents to be €435.3M per year. In total, we find a loss of €609.4M per year or €0.427M annually per km of bicycle lane if all bicycle lanes were removed.

In the second counterfactual, we convert the 407.0 km of protected lanes on large roads to painted lanes, while maintaining the cycle superhighway classifications. This increases the generalized cost of bicycling by 7.1% on average, which in turn induces 9.9% less bicycle trips and 13.7% less kilometers traveled by bicycle. We find that downgrading protected lanes leads to an annual loss of €44.8M of consumer surplus and an annual loss due to health and accidents of €127.8M. In total, we compute an annual loss of €172.6M€ or €0.424M per km of protected bicycle lane.

The third counterfactual removes the existing and planned cycle superhighway classifications. We interpret this as representing the effect of no longer having long, connected bicycle routes. The change involves 333.7 km of cycle superhighway routes and leads to an average increase of 12.8% in the generalized cost, which induces a 16.8% decrease in the number of trips and 22.8% decrease in the number of kilometers traveled by bicycle. Removing the route-level features that constitute the cycle superhighways is associated with an annual total loss of €288.1M or €0.864M per lane km.

In all three counterfactuals, we find that the total distance traveled by bicycle responds relatively more than the number of trips. This means that the number of long bicycle trips responds more than the number of short trips, in line with our observation that the demand elasticity increases with the trip cost.

## Discussion

We find a substantial impact of the provision of bicycle-relevant infrastructure on the generalized cost and the volume of cycling. We work at a very fine level of resolution, which allows us to distinguish between a large number of infrastructure types. This is first-order important as we find a difference in the generalized cost of cycling of more than a factor eight between the best and the worst infrastructure types. Thus, the type and the location of infrastructure are very important.

The counterfactual simulations performed in this study illustrate the effect of broad changes to the bicycle network, holding everything else constant. These results may be of interest when considering the consequences of expanding the bicycle network in cities with less bicycle infrastructure than Copenhagen. Our simulations show that the existing bicycle network in Copenhagen has led to a substantial increase of about 90% in distance traveled by bicycles. Interpreting these results, it should be kept in mind that our demand model comprises a large number of constants, one for each origin and each destination. These constants set a level for the total volume of bicycle traffic in and out of each destination from which the demand function predicts changes depending on the generalized cost of bicycling. This specification allows us to focus on the demand function, while remaining agnostic about the underlying mechanisms driving overall demand. The counterfactual simulations vary only the generalized cost of bicycling, and the effect on demand occurs solely through the demand function, while the constants are unchanged. As a consequence, the counterfactual simulations do not account for adaptations in location patterns and in the overall propensity to use bicycle induced by network changes. Therefore, we interpret the changes as representing short-term effects. In the longer term, location patterns and the overall propensity to bicycle can be expected to adapt to improvements in the bicycle network, which would make long-term effects larger than the short-term effect. In particular, we would expect a larger effect than a 90% increase in distance traveled by bicycle if a city comparable to Copenhagen would install a bicycle network from scratch. Previous research supports the broad conclusion that bicycle infrastructure induces more bicycle traffic e.g., refs. 2, 13, 14, and 15.

The present paper advances the literature in several ways. We find a fine-grained representation of the different infrastructure types to be very important as there are large differences in how attractive they are to cyclists. Many previous studies have not observed bicycle trajectories, which makes them less able to account for differences between infrastructure types. Relative to before-and-after analyses of specific new infrastructure projects e.g., refs. 15–21 and cross-sectional studies based on macrolevel network attributes, (e.g., refs. 22–31), we moreover gain the ability to distinguish between new induced traffic and traffic that is diverted from elsewhere (32). Some studies have used traditional transport models, (e.g., refs. 33–35), which allows them to analyze detailed counterfactual scenarios without having access to observed route choice data. In contrast, we use a large dataset of observed bicycle trajectories to estimate a model that takes the complete network into account at a very high resolution. Our findings demonstrate that this resolution is vital for understanding the impact of infrastructure on the demand for cycling.

From the counterfactual scenarios, we have calculated the net benefit of bicycle lane provision associated with the change in generalized cost, health, and accidents to be €420k to 440k per lane km per year. According to ref. 36, construction costs are in the range €0.5M to 1.5M per lane km.^*^ The estimated benefit associated with cycle superhighway status is greater, €860k per km per year, although it relates only to route-level features, holding link-level features constant. As construction costs are incurred once but benefits accrue year by year, these results indicate that the provision of well-located and high-quality bicycle infrastructure can easily generate a positive net present value in a standard cost–benefit analysis.

Copenhagen already has extensive bicycle infrastructure, so the effect of additional infrastructure may be smaller. On the other hand, we find a large net benefit of cycle superhighways, which may arise from having long and connected bicycle routes. This means that limited investments can potentially lead to large net benefits by improving overall connectivity of the bicycle network. Maps such as Fig. 1*B* can be used to identify candidate locations for such investments.

We have combined a very large dataset of GPS trajectories of bicycle trips and a very fine-grained representation of the bicycle-relevant network with a modeling approach that allows us to take the entire network into account. Our model can be applied to predict the effect of providing specific infrastructure in specific places. Similar analyses can be undertaken for other cities. In such analyses, however, the main obstacle is obtaining sufficient data on observed route choices similar to the Hövding dataset used in this study.

There is much scope for future research, applying the perturbed utility route choice model to bicycle data. On the bicycle front, it is of interest to estimate similar models using datasets from other cities to consolidate and extend the conclusions regarding the impact of bicycle infrastructure on bicycle demand. Another research avenue is investigating datasets sampled by different means or from different kinds of users to check the robustness of our conclusions.

On the methodological front, a general research agenda can be formulated for the perturbed utility route choice model, with a view to applications to bicycle traffic or other traffic through complex networks. The most important point here, we think, is to develop approaches that allow the estimation of the models at the level of individual routes. This would make it possible to avoid the data loss associated with the aggregation of data to the OD level and allows the inclusion of individual-level information. Another related avenue is to develop solution methods for the cost minimization problem in Eq. **1** that make it feasible to work with meta-networks where link pairs take the place of links. This would allow turn movements to be represented and hence allow taking into account, for example, the cost of left turns and crossing roads with car traffic.

## Materials and Methods

### Bicycle Data.

**Fig. 3.**
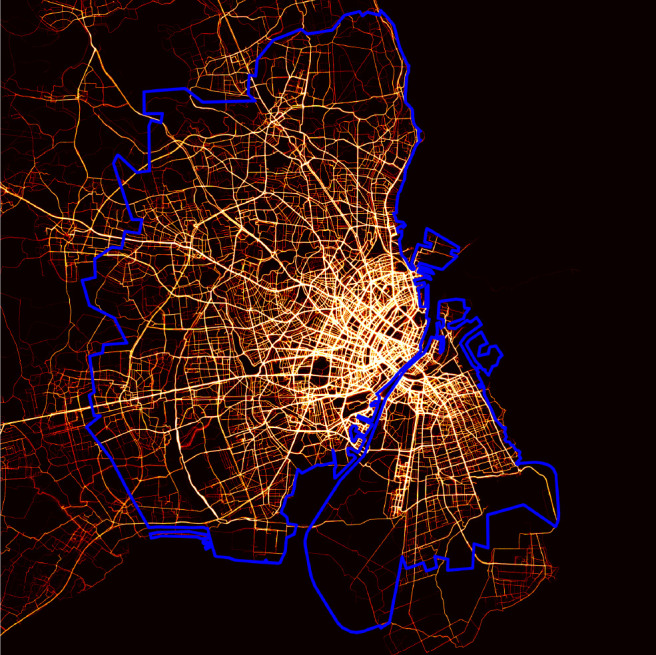
GPS traces of bicycle trips in Greater Copenhagen.

Fig. 3 shows the raw data from our sample of GPS traces of bicycle trips, collected in Greater Copenhagen (4). Fig. 1*A* presents the corresponding views of the road and dedicated bicycle network, using Open Street Map data (37). Copenhagen has an extensive bicycle network with many cycleways, especially outside central Copenhagen. In central Copenhagen, there is a dense network of protected bicycle tracks and many nonprotected bicycle lanes.^†^

*SI Appendix*, section 2C and D describes how we processed our data. In brief, the preprocessed dataset of 218,489 GPS trajectories collected from 8,588 individuals was map-matched to the Copenhagen bicycle network using software presented in ref. 38. Our estimator for the route choice model requires trips to be aggregated such that each included OD pair has at least two distinct observed routes. Therefore, we applied an algorithm that trims individual trajectories at both ends such that the trimmed trajectories have a small number of origins and destinations in common. Our analyses are based on data with 200 origins and 200 destinations, which represents a compromise between including most of the observed trajectories and avoiding many OD pairs with only a small number of observations. Robustness check with 100 and 400 origins and destinations did not indicate problems (*SI Appendix*, section 3B.3). We use the data for all OD pairs that are more than 1 km apart and have at least two distinct individual route choice observations per OD. Our estimation data comprise 152,323 trimmed trips from 7,672 individuals.

### Perturbed Utility Models.

We employ the perturbed utility route choice model (5), which is a perturbed utility model (39–41), adapted to describe the route choice through a network. A general perturbed utility model views behavior as arising from maximizing a utility over some set of possible actions, where the utility consists of a linear part and a convex perturbation function. This very general framework can represent a wide range of behavioral models (41) and, in particular, all additive random utility discrete choice models (42). For example, the multinomial logit model arises as a perturbed utility model in which the perturbation function is the Shannon entropy (43).

In the perturbed utility route choice model (5), the action for a given OD pair is a network flow, and the set of available actions is the sets of network flows that are physically consistent with a flow of mass one through the network from origin to destination.

### Route Choice Model.

A directed network comprising nodes and links (𝒱, ℰ) is described by the incidence matrix *A* with elements *a*_*ve*_ = 1 if *v* is the origin node of link *e*, −1 if it is the destination node, and 0 otherwise. A set of OD pairs ℬ is represented in terms of OD demand vectors *b* ∈ ℬ ⊂ ℝ^|𝒱|^, where *b*_*v*_ = 1 indicates the origin node of trip *b*, *b*_*v*_ = −1 indicates the destination node, and *b*_*v*_ = 0 otherwise. The flow conservation constraint *Ax* = *b* ensures that a nonnegative flow vector *x* ∈ ℝ_+_^|ℰ|^ is physically consistent with demand *b* through the network.

The perturbed utility route choice model holds that the flow vector ${\hat{x}}^{b}$ for bicyclists with demand *b* minimizes the cost function in (**1**) under the flow constraint $A{\hat{x}}^{b}=b$. Fosgerau et al. (5) show that this model generates very reasonable substitution patterns. Moreover, the model directly applies to the complete network, without a need to specify a choice set of route alternatives.

Given (noisy) observations of flow vectors, Fosgerau et al. (5) transform the active first-order conditions for the cost minimization problem to a linear regression equation that directly leads to an estimate of *β*. There is an active first-order condition for each link with positive observed flow. The transformation eliminates Lagrange multipliers corresponding to the flow conservation constraints at each node of the network. The data for the regression comprise many observations for each OD pair, which enables standard errors to be clustered by OD pair.

**Fig. 4.**
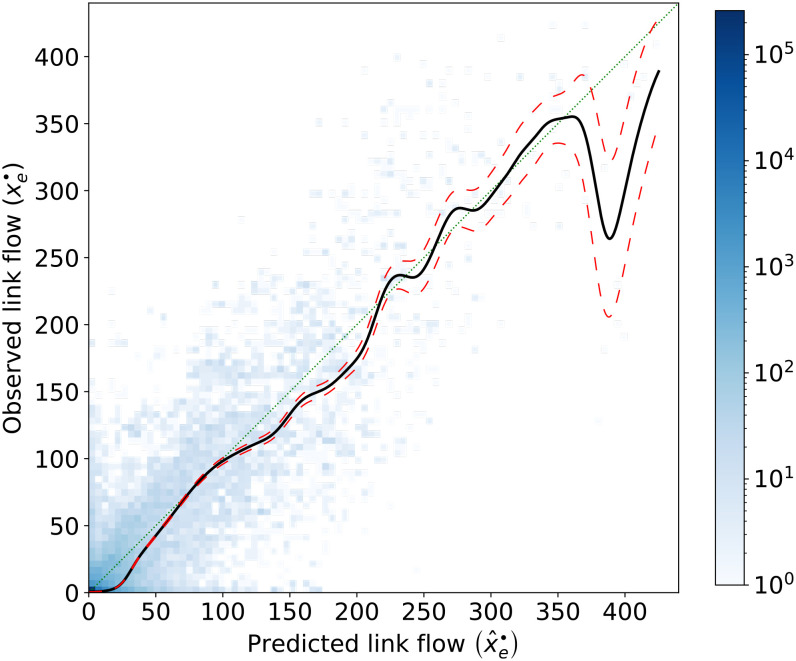
Heatmap of total observed link flow (*x*_*e*_^***⋅***^=∑_*o* ∈ 𝒪_∑_*d* ∈ 𝒟_*x*_*e*_^*od*^) against the total predicted link flow $\left({\hat{x}}_{e}^{\cdot }=\sum_{o\in O}\sum_{d\in D}{\hat{x}}_{e}^{\mathrm{od}}\right)$ for each link *e* ∈ ℰ. The color of each grid cell represents the number of links belonging to each cell. The thin green dotted line is the 45° line. The black line is a Nadaraya–Watson nonparametric regression (44, 45) with Gaussian kernel and bandwidth 10 chosen by eyeballing. The corresponding 95% pointwise confidence band is indicated by dashed red lines.

Fig. 4 plots the total observed link flow (*x*_*e*_^***⋅***^ = ∑_*o* ∈ 𝒪_∑_*d* ∈ 𝒟_*x*_*e*_^*od*^) against the total predicted link flow (${\hat{x}}_{e}^{\cdot }=\sum_{o\in O}\sum_{d\in D}{\hat{x}}_{e}^{\mathrm{od}}$) for each link *e* ∈ ℰ across all origins and destinations. A perfect prediction would exactly follow the 45° line. We find that a nonparametric regression line quite closely tracks the 45° line. This is satisfactory, especially considering that the route choice model uses only 28 parameters. The correlation (defined in *SI Appendix*, Eq. 2) between *x*^***⋅***^ and ${\hat{x}}^{\cdot }$ is $\rho (x^{\cdot },{\hat{x}}^{\cdot })=0.8894$.

*SI Appendix*, section 3B comprises a range of validation tests of the route choice model.

## Supplementary Material

Appendix 01 (PDF)

## Data Availability

The trajectory data used in this study are available from Hövding ApS but restrictions apply to the availability of these data, which were used under license for the current study, and so are not publicly available. Data are however available from the authors upon reasonable request and with permission of Hövding ApS.

## Footnotes

^*^Lower if the numbers in ref. 36 pertain to route km.

^†^*SI Appendix*, section 2A for definitions of the various infrastructure types.
