# Supplementary material for: Bikeability and the induced demand for cycling
Source: Proc Natl Acad Sci U S A. 2023 Apr 11;120(16):e2220515120. doi: 10.1073/pnas.2220515120 (PMC10120086; doi:10.1073/pnas.2220515120)
Supplement: Supplementary file 1 — Appendix 01 (PDF) [file pnas.2220515120.sapp.pdf]

www.pnas.org

Supplementary Information for

Bikeability and the induced demand for cycling

Fosgerau, Lukawska, Paulsen, and Rasmussen

Fosgerau.
E-mail: mogens.fosgerau@econ.ku.dk

This PDF file includes:

Supplementary text
Figs. S1 to S7
Tables S1 to S6
SI References

Supporting Information Text

1. Literature review

A. Perturbed utility. In a general perturbed utility model, a consumer chooses a consumption vector x from some budget set B that solves a utility maximization problem of the form $\hat{x} = \arg\max_{x \in B}(a^\intercal x - F(x))$ — that is where the utility function is a linear function "perturbed" by subtracting a convex function (1–3). Perturbed utility models are firmly rooted in modern microeconomic theory and can be interpreted as representing a population of optimizing agents whose individual behavior is described by one of a wide range of models (4). The additive random utility discrete choice model (5) belongs to the set of perturbed utility models in which the budget set B is the set of probability vectors.

(6) introduced the perturbed utility route choice (PURC) model. The model represents traveler behavior as a utility-maximizing flow across an entire network. The perturbed utility budget now requires that flow is conserved through all network nodes from origin to destination. This is a partial specification of behavior because it does not specify exactly which route a given traveler will take but only specifies the probabilities that are implied by the optimally chosen flow. A special perturbation function is specified that induces a tendency to distribute flow on more routes while allowing the optimal flow to be zero on most links in the network. (6) estimate and validate the model using 1,337,096 car trips in a large road network.

B. Bicycle route choice models. The bicycle route choice literature has almost exclusively relied on path-based models. These are random utility discrete choice models that view the route choice as a discrete choice among a predefined set of path alternatives (7–19). However, predefining the choice set is problematic, as it leads to bias in the parameter estimates (20) and risks excluding the actually chosen alternatives. This is a problem, in particular, for bicycle route choice, as bicycle networks tend to be very fine-grained (11). Researchers have been forced to discard almost half of the observed trips owing to inadequate similarities of observed trips with any of the predefined alternatives (21).

The recursive logit model (22) and the nested recursive logit model (23) view the choice of a path as a Markov chain of link choices, where the traveler at each step anticipates the expected utility associated with reaching the next node. The recursive logit models incorporate the entire network and thereby avoid pre-defining choice sets. However, a downside of this feature is that the models distribute positive flow on all network links, whereas in reality most of the network will be unused for any given origin-destination (OD) pair. (24) have applied recursive logit models to bicycle route choice, but the computation time was 15 days for the nested recursive logit model and 43 hours for the recursive logit model. (21) were not able to obtain identification with recursive logit models. An inherent limitation of these models is that computation times are strongly affected by the size of the network. The network in (24) has $42,000$ links, so the estimation of a recursive logit model for the network with $420,000$ links used in the present study would most likely not be feasible.

In this study, we instead model bicycle route choices using the PURC model, which has a range of attractive features. The model does not require choice sets to be pre-specified; it simply incorporates the entire network. In contrast to the recursive and nested recursive logit models, the PURC model leaves most of the network unused for any OD. The model allows very fast estimation of link cost parameters using linear regression, even when considering a large number of link-level variables. The model also allows estimation on much larger datasets (we estimate our model on more than 150,000 trips within a minute). Our estimating method using the PURC model requires the pre-processing of data, which leads to some loss of data; nevertheless, we are able to retain 70% of the observed route choices (see Section 2D), which is more than the choices retained with path-based approaches.

C. Bicycle infrastructure and the demand for bicycling. (25–27) review the empirical evidence on the effect of bicycle infrastructure on bicycle demand. The evidence supports that the infrastructure mostly does affect cycling demand and that the effect is heterogeneous across infrastructure types (25). (27) points out that previous studies are mostly case-based, and (26) calls for studies that use individual-level data to assess the influence of the entire network on bicycle demand.

Going into the individual studies, one group of studies reviewed by (28) and (29) conducts case-based before-and-after analyses of the demand effects of specific new bicycle infrastructure projects, using traffic count data or travel surveys (e.g., 30–35). Such studies are directly aimed at identifying the causal effects of new infrastructure. They find that demand increases up to 140% on the specific new pieces of infrastructure. Owing to the research design, however, these studies cannot distinguish between traffic actually induced by the new infrastructure and existing traffic that is attracted from elsewhere (29). In the present study, we clearly distinguish between these two mechanisms. These studies are also less suited for identifying the effects of the specific attributes of the new infrastructure, which the present study finds to be very important.

A second group of studies relates bicycle demand to the attributes characterizing entire networks at the macro-level (36–45). These studies are cross-sectional, either at the city or country level (36–38, 40, 44) or at the level of areas within a region (39, 41–43, 45). The general findings from these studies are that the overall bicycle demand is positively correlated with the length of the bicycle network (36–39, 41, 42, 44) and is negatively correlated with the share of large roads (41, 44). Both findings are supported by our study, in which we further find that the preference against large roads more than disappears when the roads are equipped with protected bicycle lanes.

(46) consider the effects of provisional pop-up bicycle lanes on travel demand during the COVID-19 pandemic in a cross-section of European cities. They find that the pop-up bicycle lanes induced an average increase in cycling of 41.6%, some of which may be new bicycle traffic while some may just be diverted. The effect of pop-up lanes was lower in cities with a larger pre-existing bicycle network per capita. For comparison, in our counterfactual simulation wherein all bicycle lanes are removed,

we find that the number of bicycle trips decreases by 37%. The research design in (46) arguably allows the identification of causal effects but cannot account for network-wide effects or explicitly for the characteristics of the existing infrastructure.

A third group of studies relates the bicycle modal share at the level of OD pairs to corresponding distances based on shortest paths (47, 48). Their results broadly agree with ours, but in contrast to the present study, they cannot take the quality of the bicycle-relevant network into account.

Finally, a fourth group of studies uses traditional transport models (49–51) or agent-based transport simulation (52) to model bicycle flows in a network. This allows them to analyze detailed counterfactual scenarios without having access to observed route choice data. (50–52) found an increase of 18%-35% (Patna, India) and 4%-9% (Copenhagen, Denmark) in bicycle use resulting from building/expanding the network of cycle superhighways. In our study, we find a similar effect in a counterfactual where the number of bicycle trips drops by 16.8% when the existing and planned cycle superhighways are removed. (49) finds a generalized cost elasticity of demand of -0.7, whereas the present study finds a higher elasticity in the range $(-1.39, -1.08)$. Equipping all links with bicycle lanes leads the demand to increase by 20% in (49), whereas we find demand to decrease by 37% when we remove all bicycle lanes.

In this study, we estimate a combined bicycle route choice model and demand model. Our model is firmly rooted in data; it incorporates a very large dataset comprising more than 150,000 observed bicycle trips across a large network and includes an extensive set of explanatory variables. From the observed route choices, we determine a generalized cost measure that is used to predict bicycle demand across the network. This improves on previous studies on a number of points. In particular, we can incorporate all bicycle infrastructure in the network and not just a few specific cases of new infrastructure. We can also assess the effects of detailed and network-wide counterfactual changes to the bicycle network, distinguishing betwen new and diverted bicycle trips. Our generalized cost measure integrates all our observed infrastructure attributes across the network to the extent that they affect route choice. The model can thus take into account both the quality of bicycle infrastructure and its location.

2. Data and data processing

A. Network data. The network representation is based on Open Street Map (OSM, 53) and includes the bicycle-relevant infrastructure — that is all network links where riding or carrying a bicycle is possible, including the elements listed in Table S1. In the representation, bicycling in both directions is allowed on all network links, while keeping track of the direction. The resulting network representation of the Copenhagen Metropolitan Area contains a total of 420,973 directed links and 324,492 nodes.

We define infrastructure types by combining three infrastructure attributes: road type (based on OSM tags), road size (based on the number of car lanes), and type of bicycle infrastructure (whether it is present and if so, whether it is a protected or a painted bicycle lane). This creates 16 distinct infrastructure types, as shown in Table S1.

The OSM network attributes have been enriched with information on land use and elevation. The land use information is obtained from an external shapefile layer (54), and includes the following categories: green areas (including green restricted areas, parks, and forests), areas near water, industrial areas, open landscape areas, low-rise urban areas, and high-rise urban areas (merged with the city center). For each directed link, the land use on the immediate right-hand side of the link is determined, tracking the length of each land use category. The elevation gradient is computed with 10 m splits of the network. Using overlay analysis, elevation information per 10 m is attributed to each link. Based on this, the slope and difference in elevation are obtained per 10 m on each link, and the total vertical meters gained when the slope is greater than 3.5 % per direction per link are determined.

B. Trajectory data. The data were collected in Greater Copenhagen (the study area framed in Figure S1) between September 16, 2019 and May 31, 2021 from 9,564 individuals using a Hövding head protection airbag helmet designed for cyclists (55). Positional data were passively collected among users who had given consent to share their data and were transmitted to a database server through the users' smartphones connected to the airbag helmet via Bluetooth. The dataset of observed trajectories contains 347,430 trips starting and/or ending in Greater Copenhagen and covering a total of 939,711.8 km traveled by bicycle. Each trip connects an OD pair, represented by the starting point and the endpoint of the trip, respectively.

Figure S2 compares the age distribution of Hövding users (provided by the company, 55) to data from the Danish national travel survey (56), where we have selected individuals above 15 years old, interviewed in the period 2019-2021, living in the Greater Copenhagen Area, and who have reported at least one bicycle trip. This comparison suggests that the Hövding data somewhat underrepresents the age groups 15-24 years and above 65 years. The distribution of the Hövding sample on gender (m/f: 41.4/58.6%) is similar to that of the travel survey (m/f: 45.4/54.6%).

C. Map matching. Each of the observed trajectories has been map-matched to the bicycle network using the hidden Markov algorithm proposed in (57). The algorithm allows for off-road parts in the matched route, which are often necessary for bicycle trips, as bicyclists do not always stick to formal roads and paths. However, our network has a high resolution, and we found that only 35 trips were matched with off-road segments. We discarded these trips from the subsequent analysis.

D. OD data and trip trimming. All trips shorter than 1 km were discarded. Furthermore, we discarded circuitous routes that were more than $\frac{\pi}{2}$ times longer than the crow-fly distance. Trips with loops, where a part of the route was repeated or where the same network node was visited twice, were also discarded. Finally, we discarded trips where the map matching algorithm failed to match the entirety of the trip. The resulting dataset after the filtration steps comprised 218,489 trips from 8,588 individuals covering a total of 762,791.8 km.

Our estimator for the PURC model requires multiple observations in each OD pair. Because common ODs are very rare in a large network, we follow (6) and trim the observed trips such that the trimmed trips share common ODs. Our algorithm selects first a set of origins and then a set of destinations. A trip is included in the estimation data if it passes first a selected origin and then a selected destination, and only the part of the trip between the selected origin and destination is included.

More specifically, we include origins one by one, choosing in each step the origin that maximizes the total length of trips that it allows to include, while trimming the additional included trips to begin from that origin. After a list of origins has been compiled, we find in a similar way a list of destinations. The final output is a long list of origins and destinations. Our main results are obtained using 200 origins and 200 destinations.

To ensure that the generated origins and destinations are all found within Greater Copenhagen (see Figure S1), in this step, we only consider trips that both start and end within Greater Copenhagen. This filtration makes the dataset used for this task slightly smaller than the final estimation dataset, where we require only that either the origin or the destination is within Greater Copenhagen (see Section 2B). The dataset used for finding origins and destinations comprises 208,410 trips (703,837.5 km) across 8,456 individuals.

After compiling the list of origins and destinations, we identify, for each trip, the first origin and the last destination that are on the list. Only the trips that include first an origin and then a destination from the list are included. Included trips are trimmed to begin and end at these points.

This process increases the likelihood of the included trips having origins and destinations in common with other included trips. Data are lost if the number of origins and destinations is small, which speaks for including many origins and destinations. However, the estimator used combines trips that are matched using the same OD pair into an observed average flow vector for that OD pair. Increasing the number of origins and destinations means that the observed average flow will be based on fewer matched trips per active OD pair, implying more noise. It also means that there will be more unmatched trips, trips that are alone in using an OD pair, and these trips cannot be used for estimation. Therefore, we carefully choose the number of origins and destinations to balance these concerns.

At this stage, the data include trips that are not matched to another trip with the same OD. Therefore, we extend the algorithm to reduce the number of such trips. The algorithm extension first identifies the longest unmatched trip. This trip is then gradually trimmed by trying combinations of later origins (from the list of candidates) and prior destinations (from the list of candidates) until it is found to travel between an origin and a destination that matches another trimmed trip, matched or unmatched. Trips that fail to find a match are discarded. The algorithm continues with the longest remaining unmatched trip until all unmatched trips have been either matched or discarded.

The number of origins and destinations on which to base the trip trimming was selected so as to maximize the number of OD pairs that have at least ten observed trips (after recovering unmatched trips). This was obtained when using 200 origins and 200 destinations. As a check, we also report estimation results from the route choice model with 100 and 400 origins and destinations (Table S5). The parameter estimates are not very sensitive to this change, as we shall see in Section 3B.3.

Table S2 summarizes the size of the datasets after the main steps of data processing.

In conclusion, we retained 70% of the observed trips for the estimation. This is much higher than seen in traditional path-based route choice studies (58, 59). Figure S1 shows the heat maps of the trajectory data after the initial data filtration (a), the trips connecting candidate origins and destinations (b), and the trimmed trips used for estimation (c). We observe that the trimmed trips preserve a good coverage of the network.

E. Computing predictions. The predicted flow for a trip starting in $o \in \mathcal{O}$ and ending in $d \in \mathcal{D}$ is the flow vector $\hat{x}^{od}$ that minimizes the cost

$$C(x) = \sum_{e \in \mathcal{E}} l_e \left(c_e x_e + F(x_e) \right), \quad [1]$$

subject to the flow conservation constraint. The generalized cost association with this OD pair is $C(\hat{x}^{od})$.

The edge cost rates $c_e, e \in \mathcal{E}$ are found by multiplying the corresponding row in the link attribute matrix Z_e with the $\hat{\beta}$ parameters estimates reported in Table S3, i.e., $c_e = Z_e \hat{\beta}$. The minimization problems are solved using conic optimization in the software Mosek Fusion (60). We define the average length between o and d corresponding to the predicted flows as $\hat{l}^{od} = \sum_e \hat{x}_e^{od} l_e$. Finally, we obtain the predicted average generalized cost between o and d, omitting the perturbation term, as $\hat{c}^{od} = \sum_e c_e \hat{x}_e^{od}$.

3. Route choice model

A. Estimation results. Table S3 shows the estimated parameters for the preferred model specification, along with clustered heteroscedasticity consistent standard errors. To aid interpretation, the last column of the table shows the parameters divided by the parameter for the constant; thus, the scaled parameters express the generalized cost rate in terms of metres traveled on the reference category road. We discuss the results in terms of the scaled parameters.

The reference category is residential roads without specific bicycle infrastructure in low-rise urban areas. The parameter for the constant thus represents the generalized cost of traveling 1 m by bicycle on the reference category.

The next set of parameters measures the impact of various mutually exclusive infrastructure types on the link cost rate. Links with stairs (intended for pedestrians) are classified as a separate category and incur a penalty of 76%. The cost rate is

up to 27% higher for infrastructure types that are shared with pedestrians. The additional cost rate for "living streets" is not significantly different from zero.*

Compared with the reference, bicyclists have some preference against large roads (roads with at least two lanes in one direction, 11%), whereas the preference against medium roads (roads with at most one lane in each direction) is small and statistically insignificant.

Provision of dedicated bicycle infrastructure quite substantially reduces the generalized cost of bicycling. Cycleways (bicycle paths in own trace) have 20% lower cost rate than the reference. On residential and medium roads, bicycle lanes, whether protected or just painted, reduce the cost rate by 14% and 22%, respectively. The type of bicycle lane has a significant effect on the cost rate for the large roads category: painted bicycle lanes have only a small and statistically insignificant effect on cost rate, whereas protected bicycle lanes reduce the cost rate by 34%. It makes clear intuitive sense that the impact of bicycle lanes is larger the larger the road, and that only protected lanes affect the largest roads where car traffic is heavier.

Provision of bicycle-friendly infrastructure thus has a substantial effect on route choice. As evident from Section "Bicycle travel demand"in the main text, this translates into a substantial effect on the volume of bicycle trips.

A number of routes are marketed as so-called cycle superhighways. This label is applied to high-quality, continuous bicycle routes built to cater to commuter cyclists. The cost rate on the links of these routes is 12% lower than that on similar links without the cycle superhighway label. The cycle superhighway label and the associated infrastructural changes are likely the cause of the reduction in generalized cost. However, the cycle superhighway label likely has just been attached to routes that were already attractive. To check this, we include a variable indicating routes that are proposed to become cycle superhighways in the future. We find that the cost reduction associated with these links is almost exactly the same as the cost reduction found for the actual cycle superhighway links. The model already accounts for a range of link characteristics, including upgrades to the bicycle infrastructure that take place in the process of creating a cycle superhighway. The attraction of the actual and proposed cycle superhighways could therefore be attributable to route-level and not link-level features; perhaps the feature that these routes are high-quality, continuous bicycle routes (61, 62). The similarity of the parameters suggests that not the labeling but rather the fact that already attractive routes have been selected to receive the cycle superhighway label makes the difference.

The next set of parameters accounts for the land use near cycleways. We treat cycleways separately, as they turned out to act different from the other infrastructure types. The cost rate is much reduced for cycleways in industrial areas (48%) or green areas (53%) compared to low-rise urban areas. It makes intuitive sense that cycleways in green areas may be pleasant. Another potential explanation that also applies to industrial areas is the attractiveness of isolation from heavy traffic. The parameters for cycleways near water or open landscape are not statistically significant.

For the other infrastructure types, the generalized cost is lower near all other land uses than low-rise urban areas, but the differences are not statistically significant for all land use types. The largest cost reduction is found for links near water (26%).

The last set of parameters concerns some special link characteristics. The elevation gain variable measures the total elevation gain on a link that has a gradient of 3.5 percent or more. It aggregates the vertical distance on the parts of the links where the slope is at least +3.5%. The scaled parameter is estimated to be 16.6, which means that an elevation gain of 0.05 me per meter implies an increase of 83% in the cost rate, which seems reasonable. If the surface is gravel, the cost rate increases by 19%, whereas the parameter for cobblestones is small and not statistically significant. Finally, going against (car) traffic on one-way streets increases the cost rate by 59%.

B. Model validation.

B.1. Comparison of observed and predicted flows by infrastructure type. Using the estimated parameters, we compute the predicted flows for each OD pair. Table S4 compares the observed and predicted flows, showing the percentage of flow that occurs on each link type. For comparison, the first column of the table shows the corresponding shares weighted just by link lengths, which is what would be the result if traffic was distributed on the network at random. We find, supporting the model, that the observed and predicted flow shares are very similar. They are both very different from the network shares, especially because cyclists use roads with bicycle infrastructure much more than would have been the case if traffic was randomly distributed on the network.

B.2. Comparison of observed and predicted flows by OD pair. To compare the observed and predicted flow vectors for a given OD pair, we introduce a correlation measure as follows: defined for convenience as

$$\mathbb{E}_l(x) = \sum_{e \in \mathcal{E}} \frac{l_e}{\sum_{e' \in \mathcal{E}} l_{e'}} x_e.$$

We then compute the correlation between the observed and predicted flow on a random kilometer of road (where $\circ$ is the Hadamard product):

$$\rho^{od} = \rho(x^{od}; \hat{x}^{od}) = \frac{\mathbb{E}_l(x^{od} \circ \hat{x}^{od}) - \mathbb{E}_l(x^{od})\mathbb{E}_l(\hat{x}^{od})}{\sqrt{\mathbb{E}_l(x^{od} \circ x^{od}) - (\mathbb{E}_l(x^{od}))^2}\sqrt{\mathbb{E}_l(\hat{x}^{od} \circ \hat{x}^{od}) - (\mathbb{E}_l(\hat{x}^{od}))^2}}. \quad [2]$$

The correlation is bounded between -1 and 1, with 1 corresponding to perfect correlation.

* Small residential streets with parked cars and no bicycle infrastructure, where there may be children playing etc.

We compute the correlation for every OD pair in the data. Figure S3 plots the correlation against the number of observed trips in each OD pair and against the expected route length in each OD pair. We find that the mean correlation ranges from more than 0.5 to more than 0.75. The correlation increases with the increase in the number of observed trips, which is reasonable since sampling noise causes the observed flow to differ from the predicted flow and hence decreases the correlation. The correlation decreases with route length, which may also be attributable to sampling noise because the number of observed trips decreases with trip length.

The average correlation across all the OD pairs used for the route choice estimation is found to be 0.6356. When weighting by the number of observed trips, to obtain the correlation between the observed and predicted link flows for an average observed trip, the correlation is 0.7001.

B.3. Changing the number of ODs. As mentioned in Section 2D, the number of origins and destinations needs to be chosen prior to the estimation. As a robustness check, we estimate the model using 100 and 400 Os and Ds in addition to the 200 used for the main result. Table S5 shows the estimates, which are broadly similar across models.

B.4. Cross-validation. Because we use a very large dataset with more than 1.8 million rows in the linear regression equation, our model is not very likely to suffer from over-fitting. This expectation is confirmed by a test where we randomly split the OD pairs into two separate datasets (data split A and data split B). Data split A comprises 5,520 OD pairs with 929,569 rows for the linear regression, and data split B comprises 5,519 OD pairs with 937,198 rows for the linear regression. Table S6 shows the parameter estimates from the route choice model for the two data splits A and B, with the estimates from the model based on the full sample included for easy reference. The parameter estimates appear to be quite stable across splits.

As an out-of-sample prediction test, we compute the predicted flows for each sample split, based on the parameters estimated for the other sample split. Figure S7 plots the observed flows vs. the predicted flows for each split combination as well as for the full sample. We find that the out-of-sample fit is very similar to the in-sample fit. The correlations between the observed and out-of-sample predicted link flows, computed according to Eq. 2, are 0.879 and 0.889 for the models based on data split A and data split B, respectively. This is comparable to the correlation of 0.889 found for the full sample model.

We similarly calculate correlations between the observed and out-of-sample predicted flows for each OD pair. The average OD link flow correlation across OD pairs is found to be 0.629 and 0.638 (0.691 and 0.705 when weighting by number of observed trips), respectively, when the models based on data split A and data split B are used out-of-sample. This is very similar to the correlation value of 0.634 (0.699 when weighted by number of observed trips) found for the overall model applied in-sample.

4. Gravity model

A. Specification of the demand function. The gravity model assumes that the demand is driven by the cost $\hat{c}^{od}$ from the route choice model and not just by the OD distance. To test whether this is the case, we split the cost into a length component and a residual quality component that is orthogonal to length. In this decomposition, we omit the perturbation term, as this term is expected to increase with a decrease in the OD distance because shorter trips have fewer relevant routes. For the test, we therefore compute the length component as the predicted value in a linear regression of the cost $\hat{c}^{od}$ while excluding the perturbation term against the predicted trip length. The quality component is the residual from this regression. We have then estimated the gravity model in Eq. (3) with two demand functions, one for length and one for quality. As before, both are specified as piece-wise linear.

$$\ln E\left[Y^{od}\right] = D_1\left(length^{od}\right) + D_2\left(quality^{od}\right) + \delta + \eta_o + \gamma_d \qquad [3]$$

Figure S4 shows in blue the estimated demand function from the original model (Eq. (2) in the main text) along with the demand functions from the model in Eq. (3), where the orange dotted curve is the influence of length on demand and the green dotted curve is the influence of quality on demand. We observe that the curve of the influence of quality is least as steep as the curve of the estimated demand function from the original model. We therefore conclude that collapsing the two effects (length and quality) into a single demand function yields a conservative estimate of the effect on demand.

B. Demand elasticity. The elasticity of demand is the relative change in demand per relative change in cost. Thus, an elasticity of -1 implies that a 10% increase in cost leads to a 10% decrease in demand. Figure S5 shows the elasticities calculated along the estimated demand curve. The result indicates that the elasticity decreases about monotonically with the cost.

5. Counterfactuals

A. Method. Using the average predicted generalized OD costs $\hat{c}^{od}$, the estimated function D, and values of δ, η_o, and γ_d, we apply the estimated gravity model to compute $\hat{Y}^{od}$, the predicted number of trips between any $o \in \mathcal{O}$ and $d \in \mathcal{D}$. By weighting each OD flow prediction with the corresponding predicted average length between o and d, we find the total sum of predicted kilometers traveled in our sample. This number corresponds to the size of our sample of bicycle trips. We scale it to the actual annual number of kilometers traveled by bicycle in the study area. We can estimate this roughly to be 1,026 million km, computed using (63) as the total kilometers in Frederiksberg and Copenhagen municipalities, and 50% of the kilometers in the suburbs. In this way, we find that each predicted trip represents $\zeta = 1{,}943$ annual trips.

When simulating a counterfactual s, we first adjust the link attribute matrix Z according to the counterfactual scenario and then obtain a new link attribute matrix Z^s, and corresponding link-specific costs rates $\tilde{c}^s_e = Z^s_e \hat{\beta}$ (visualized for our three

counterfactuals in Figure S6). Using these new link cost rates, we recompute the cost minimizing flows, which we denote as $\hat{x}^{od,s}$. We also compute the average predicted generalized OD costs $\hat{c}^{od,s} = \sum_{e\in\mathcal{E}} l_e \hat{\hat{x}}_e^{od,s} c_e^s$. We keep the estimated δ, η_o, and γ_d from the base scenario, and scale each trip with the same factor ζ as in the base scenario.

The relative cost increase in counterfactual s compared with the base scenario is

$$\frac{\sum_{o\in\mathcal{O}}\sum_{d\in\mathcal{D}} \hat{Y}^{od} \frac{\hat{c}^{od,s}-\hat{c}^{od}}{\hat{c}^{od}}}{\sum_{o\in\mathcal{O}}\sum_{d\in\mathcal{D}} \hat{Y}^{od}}. \quad [4]$$

The relative decrease in the number of trips in counterfactual s relative to the base scenario is

$$1-\frac{\sum_{o\in\mathcal{O}}\sum_{d\in\mathcal{D}} \hat{Y}^{od,s}}{\sum_{o\in\mathcal{O}}\sum_{d\in\mathcal{D}} \hat{Y}^{od}}. \quad [5]$$

The corresponding measure for the number of kilometers traveled is

$$1-\frac{\sum_{o\in\mathcal{O}}\sum_{d\in\mathcal{D}} \hat{Y}^{od,s}\hat{l}^{od,s}}{\sum_{o\in\mathcal{O}}\sum_{d\in\mathcal{D}} \hat{Y}^{od}\hat{l}^{od}}. \quad [6]$$

To compute the consumer surplus (64), we first convert the generalized cost to length units by dividing the it with the constant term in $\hat{\beta}$ (0.4555). Next, we use the average speed in our sample (14.84 km/h) to convert the length units into travel time units. The change in travel time then becomes

$$\Delta T^{od,s} = \frac{1}{14.84}\frac{\hat{c}^{od,s}-\hat{c}^{od}}{0.4555}. \quad [7]$$

The value of time for cyclists in Denmark is 16.13 € per hour according to the official guidelines (65). Combining the change in travel time, the value of time, and the change in demand, we apply the rule of a half to compute the change in consumer surplus for each counterfactual scenario:

$$16.13\cdot\zeta\cdot\sum_{o\in\mathcal{O}}\sum_{d\in\mathcal{D}} \Delta T^{od,s}\frac{\hat{Y}^{od}+\hat{Y}^{od,s}}{2}. \quad [8]$$

Health and accident benefits are computed multiplying the combined unit price of 0.91 € (1.17 € for health and -0.26 € for accidents (65)) by the scaled change in kilometers traveled to get the monetary annual benefit

$$0.91\cdot\zeta\cdot\sum_{o\in\mathcal{O}}\sum_{d\in\mathcal{D}} \hat{Y}^{od,s}\hat{l}^{od,s}-\hat{Y}^{od}\hat{l}^{od}. \quad [9]$$

The societal benefit owing to cyclists' travel time, health, and accidents is the sum of the consumer surplus benefits and the health/accident benefits. The societal benefit per changed network length is determined by dividing the societal benefit with the sum of the (directed) link lengths of the links that were changed to create the counterfactual s. However, for the third counterfactual on cycle superhighways, the corresponding route distances were used instead of the network length, as the expense related to this counterfactual occurs at the route level.

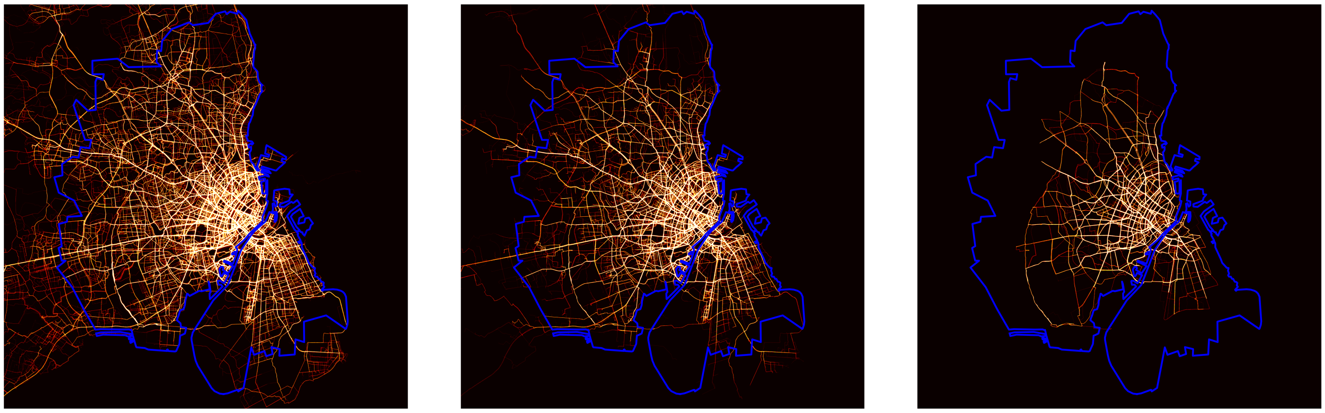

(a) After initial data filtration

(b) Trips connecting candidate Os and Ds

(c) Trimmed trips used for estimation

Fig. S1. Heat map of anonymized GPS traces after different filtration phases. Study area shown in blue. (a): All GPS points after initial filtration ; (b): GPS points of the trips used for model estimation (untrimmed filtered trips); (c): GPS points of the trips used for model estimation (trimmed filtered trips). For visualization, trips have been anonymized by removing a random number of points at their start and end.

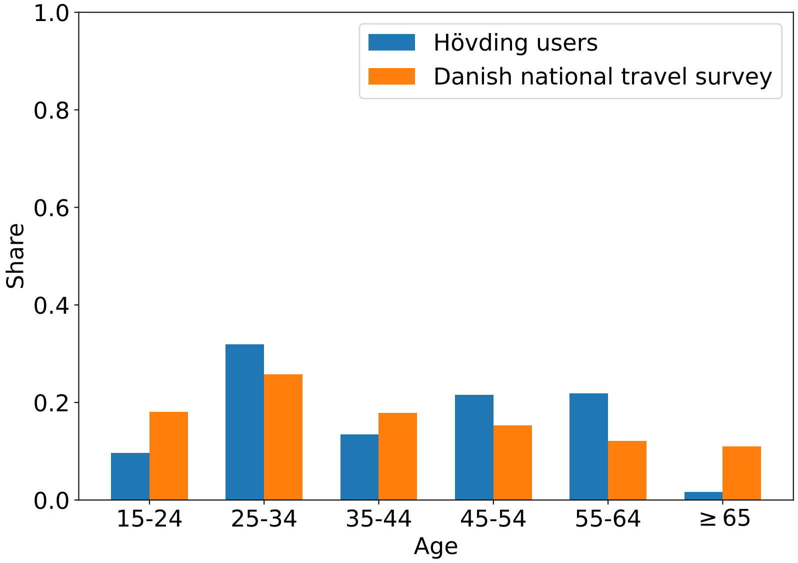

Fig. S2. Age distribution of cyclists in the Hövding sample and in the Danish national travel survey.

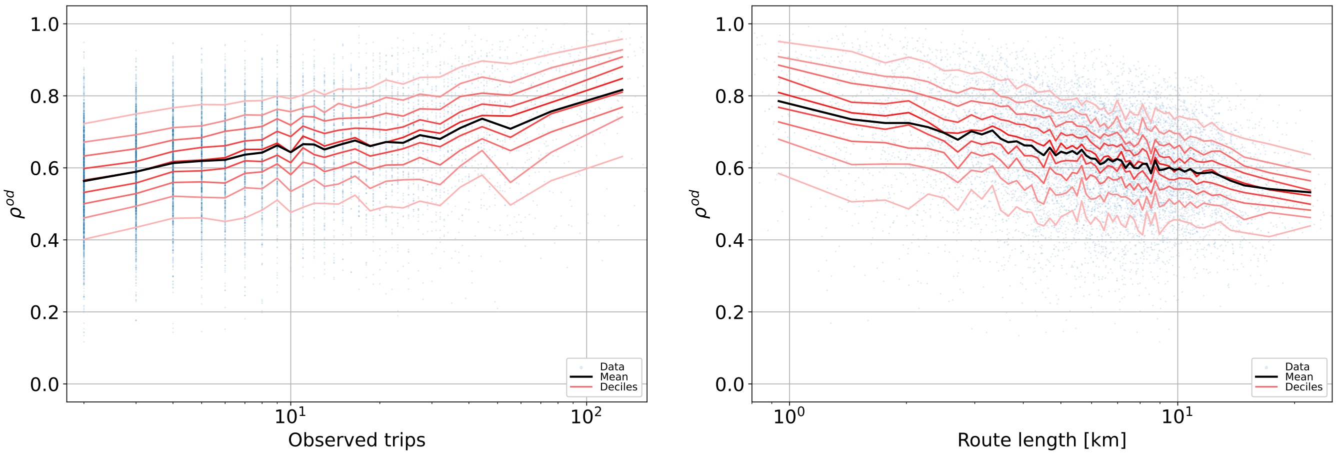

(a) Number of observed trips

(b) Expected route length

Fig. S3. Correlation between predicted and observed flow as defined in Eq. (2) plotted against the number of observed trips per OD pair (panel a) and the expected route length per OD pair (panel b). The black line indicates mean correlation, and the red lines indicate the corresponding binned deciles.

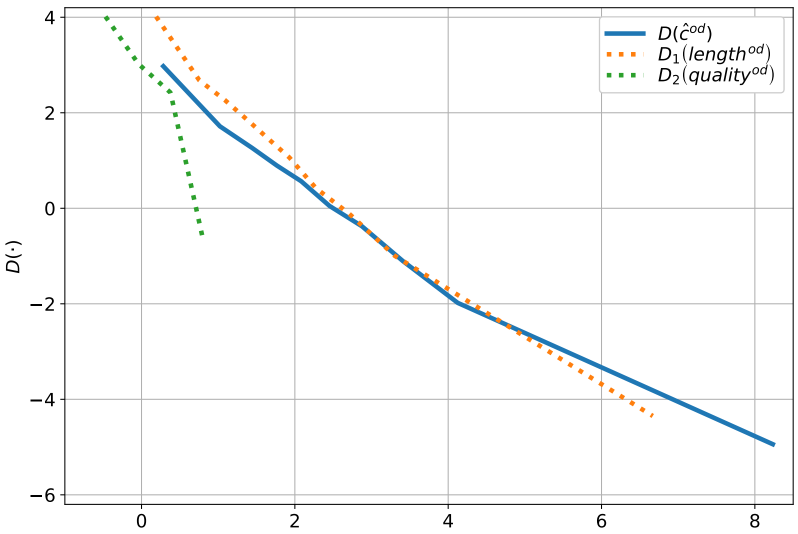

Fig. S4. Estimated piecewise linear specifications of D (from Eq. (2) in the main text) for three different explanatory variables. Blue: Demand function D from the original model (Eq. (2) in the main text). Orange & green: Demand functions D_1 and D_2 from the model in Eq. (3), respectively.

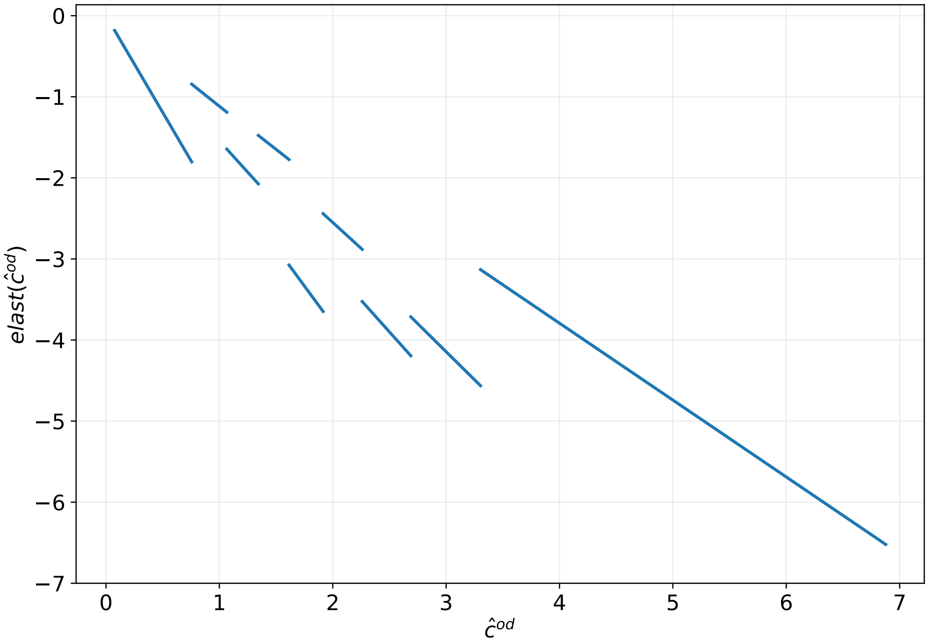

Fig. S5. Demand elasticities $(elast(\hat{c}^{od}))$ calculated from the estimated demand function using $elast(\hat{c}^{od}) = D'(\hat{c}^{od})\hat{c}^{od}$. The demand elasticity at a point $\hat{c}^{od}$ is the relative change in demand per relative change in cost.

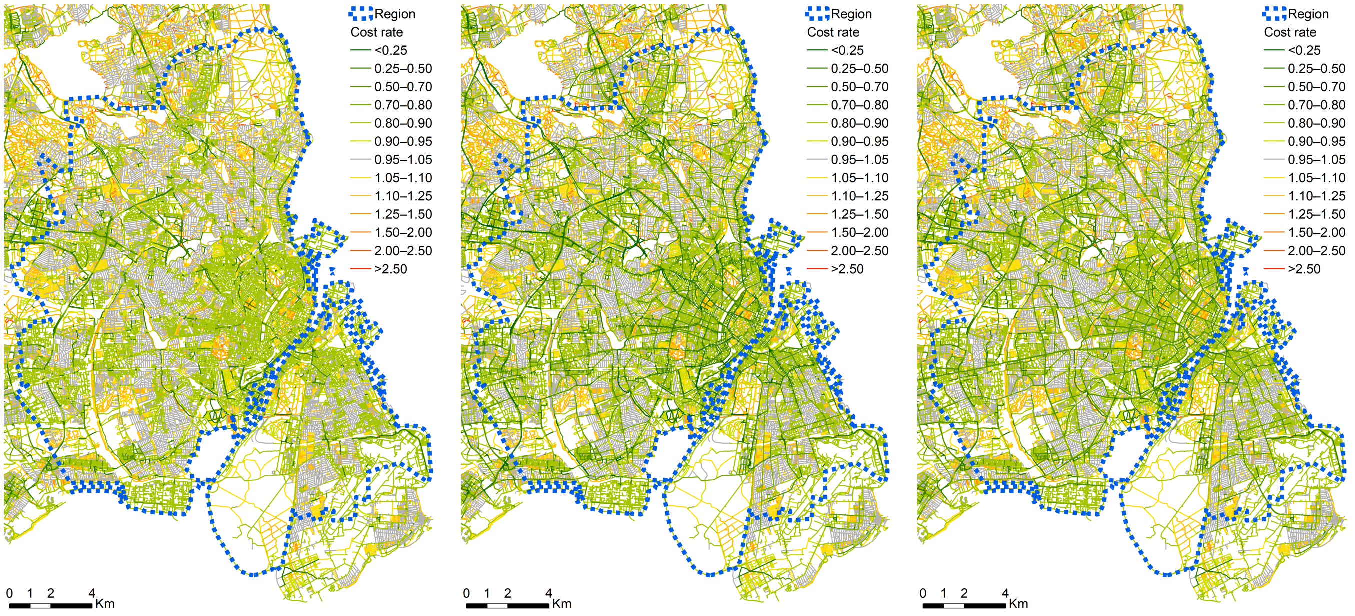

(a) No bicycle lanes

(b) Painted lanes instead of protected on large roads

(c) No existing or planned cycle superhighway classifications

Fig. S6. The estimated cost rates of links in the three counterfactual scenarios. The gray links mark the reference case: residential roads with no bicycle infrastructure in low-rise urban areas, scaled to a value of 1. Shades of green correspond to increasingly more attractive links, and shades of orange and red correspond to increasingly less attractive links.

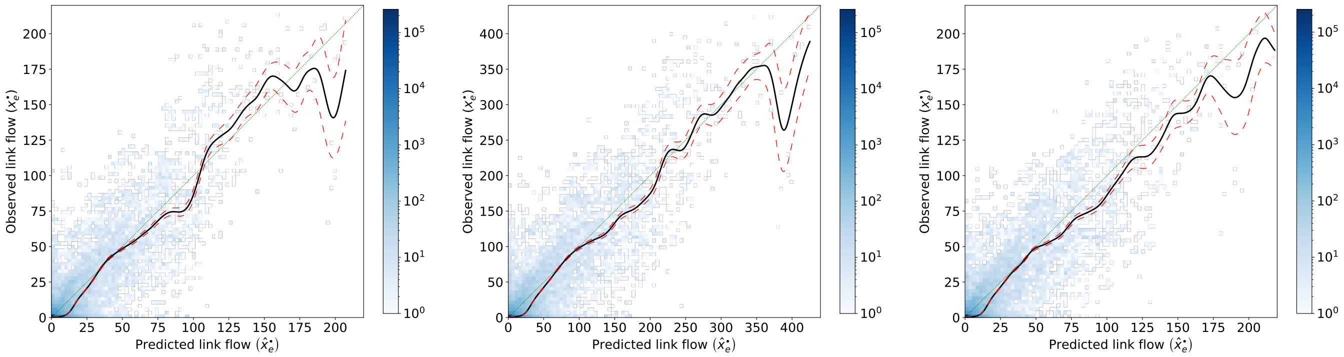

(a) Model based on data split A

(b) Model based on the full data

(c) Model based on data split B

Fig. S7. Heatmap of total observed link flow $\left(x_e^{\cdot} = \sum_{o\in\mathcal{O}} \sum_{d\in\mathcal{D}} x_e^{od}\right)$ against the total predicted link flow $\left(\hat{x}_e^{\cdot} = \sum_{o\in\mathcal{O}} \sum_{d\in\mathcal{D}} \hat{x}_e^{od}\right)$ for each link $e \in \mathcal{E}$. The color of each grid cell represents the number of links belonging to that cell. The thin green dotted line is the 45° line. The black line is a Nadaraya-Watson non-parametric regression (66, 67) with Gaussian kernel and bandwidth chosen by eyeballing (a bandwidth of 5 for data splits A and B, and a bandwidth of 10 for the full data). The corresponding 95% pointwise confidence band is indicated by dashed red lines. The model based on data split A has been applied out-of-sample on data split B and vice versa.

Infrastructure type	Description
Residential roads w/o bicycle infrastructure<br>Residential roads w/ painted bicycle lanes<br>Residential roads w/ protected bicycle lanes	Residential roads: Roads with OSM tag *highway=‘residential’*
Medium roads w/o bicycle infrastructure<br>Medium roads w/ painted bicycle lanes<br>Medium roads w/ protected bicycle lanes	Medium roads: Roads with OSM tags *highway={‘primary’,‘secondary’,‘tertiary’,‘unclassified’}*, that have at most one car lane per direction.
Large roads w/o bicycle infrastructure<br>Large roads w/ painted bicycle lanes<br>Large roads w/ protected bicycle lanes	Large roads: Roads with OSM tags *highway={‘primary’,‘secondary’,‘tertiary’,‘unclassified’}*, that have at least two car lanes in at least one direction.
Cycleways	OSM tag *highway=‘cycleway’*
Footways	OSM tag *highway=‘footway’*
Living streets	OSM tag *highway=‘living_street’*
Shared paths	OSM tags *highway={‘path’,‘track’,‘service’}*
Pedestrian zones	OSM tag *highway=‘pedestrian’*
Stairs	OSM tag *highway=‘steps’*

Table S1. Network attributes related to infrastructure type and their associated OSM tags

	Trips	Users	Kilometres
After initial data filtration	218,489	8,588	762,791.8
Trips connecting candidate Os and Ds	152,323	7,672	614,909.7
Trimmed trips used for estimation	152,323	7,672	417,358.0

Table S2. Summary of the dataset size after different filtration subprocesses. The subprocesses correspond to panels (a), (b), and (c) in Figure S1, respectively.

Parameter	Coef.	Std. err.	*P*-val.	Scaled
**Constant**	−0.456	0.027	***	1
**Infrastructure**				
Stairs	−0.342	0.096	***	0.750
Pedestrian zones	−0.121	0.025	***	0.265
Footways	−0.090	0.012	***	0.198
Shared paths	−0.026	0.016		0.057
Living streets	−0.051	0.039		0.113
Cycleways	0.089	0.037	*	−0.195
Residential roads				
No bicycle infrastructure	——-	——-	——-	——-
W/ bicycle infrastructure	0.065	0.019	***	−0.142
Medium roads	−0.005	0.018		0.010
No bicycle infrastructure	——-	——-	——-	——-
W/ bicycle infrastructure	0.101	0.018	***	−0.221
Large roads	−0.050	0.015	***	0.110
No bicycle infrastructure	——-	——-	——-	——-
W/ painted bicycle lanes	0.014	0.024		−0.030
W/ protected bicycle lanes	0.154	0.016	***	−0.338
**Bicycle route classification**				
No classification	——-	——-	——-	——-
Cycle superhighway	0.057	0.014	***	−0.126
Proposed cycle superhighway	0.055	0.012	***	−0.121
**Land use, cycleways**				
High-rise urban areas	0.119	0.041	**	−0.260
Low-rise urban areas	——-	——-	——-	——-
Industrial areas	0.221	0.052	***	−0.486
Green areas	0.243	0.054	***	−0.534
Areas near water	0.043	0.052		−0.093
Open landscape	−0.037	0.123		0.082
**Land use, other infrastructure**				
High-rise urban areas	0.088	0.024	***	−0.193
Low-rise urban areas	——-	——-	——-	——-
Industrial areas	0.072	0.038		−0.158
Green areas	0.054	0.037		−0.119
Areas near water	0.118	0.030	***	−0.259
Open landscape	0.083	0.096		−0.182
**Elevation gain, >35 m/km**	−7.559	3.682	*	16.595
**Surface type**				
Asphalt	——-	——-	——-	——-
Cobblestones	−0.014	0.019		0.030
Gravel	−0.086	0.015	***	0.189
**Wrong way**	−0.266	0.007	***	0.584
N		1,866,767		

Table S3. The estimated parameters for the bicycle route choice model in the main text (200 origins and 200 destinations). Standard errors are clustered (per OD pair) and are heteroscedasticity-consistent. The scaled values are scaled such that the parameter "Constant" has a value of 1. Significance levels are * *P*-value $\leq$ 0.001, ** *P*-value $\leq$ 0.01, and * *P*-value $\leq$ 0.05. *N* denotes the number of rows in the linear regression equation.**

	Network [%]	Observed use [%]	Predicted use [%]
**Constant**	100.00	100.00	100.00
**Infrastructure**			
Stairs	0.12	0.07	0.07
Pedestrian zones	0.19	0.72	0.35
Footways	8.24	6.80	2.49
Shared paths	38.54	6.21	6.26
Living streets	0.55	0.34	0.61
Cycleways	8.95	10.02	19.36
Residential roads	22.88	13.50	16.57
No bicycle infrastructure	22.61	10.22	12.74
W/ bicycle infrastructure	0.27	3.28	1.86
Medium roads	18.37	41.06	40.93
No bicycle infrastructure	14.34	3.07	1.90
W/ bicycle infrastructure	4.03	37.99	39.03
Large roads	2.16	21.27	18.68
No bicycle infrastructure	1.00	3.63	0.75
W/ bicycle lanes	0.08	1.32	1.19
W/ protected bicycle tracks	1.08	16.32	16.74
**Bicycle route classification**			
No classification	92.01	39.39	39.51
Cycle superhighway	2.33	23.50	23.19
Proposed cycle superhighway	5.66	37.11	37.31
**Land use, cycleways**			
High-rise urban areas	0.58	4.05	5.27
Low-rise urban areas	3.49	1.20	1.37
Industrial areas	0.64	1.45	2.62
Green areas	1.09	2.09	4.86
Areas near water	0.11	0.76	0.87
Open landscape	3.04	0.48	0.69
**Land use, other infrastructure**			
High-rise urban areas	8.25	60.07	56.60
Low-rise urban areas	36.54	9.47	10.25
Industrial areas	7.92	5.59	5.55
Green areas	16.05	8.87	7.41
Areas near water	1.02	4.93	3.24
Open landscape	21.27	1.05	1.28
**Elevation gain, $>$ 35 m/km**	0.16	0.01	0.02
**Surface type**			
Asphalt	86.25	95.85	97.62
Cobblestones	0.96	2.06	1.72
Gravel	12.79	2.09	0.65
**Wrong way**	3.54	9.15	2.17

Table S4. Distribution of length shares for various link characteristics. Network indicates the share of the network, observed use indicates the share of the observed trips, and predicted use indicates the share of the predicted flow.

Number of Os and Ds	100				200				400			
**Parameter**	**Coef.**	**Std. err.**	***P*-val.**	**Scaled**	**Coef.**	**Std. err.**	***P*-val.**	**Scaled**	**Coef.**	**Std. err.**	***P*-val.**	**Scaled**
**Constant**	−0.493	0.035	***	1	−0.456	0.027	***	1	−0.360	0.027	***	1
**Infrastructure**												
Stairs	−0.257	0.122	*	0.521	−0.342	0.096	***	0.750	−0.555	0.090	***	1.541
Pedestrian zones	−0.141	0.029	***	0.285	−0.121	0.025	***	0.265	−0.106	0.024	***	0.296
Footways	−0.073	0.014	***	0.147	−0.090	0.012	***	0.198	−0.099	0.012	***	0.275
Shared paths	−0.041	0.019	*	0.082	−0.026	0.016		0.057	−0.039	0.016	*	0.109
Living streets	−0.049	0.054		0.100	−0.051	0.039		0.113	−0.076	0.039	*	0.212
Cycleways	0.104	0.037	**	−0.211	0.089	0.037	*	−0.195	0.059	0.033		−0.163
Residential roads												
No bicycle infrastructure	—-	—-	—-	—-	—-	—-	—-	—-	—-	—-	—-	—-
W/ bicycle infrastructure	0.030	0.023		−0.062	0.065	0.019	***	−0.142	0.046	0.018	*	−0.128
Medium roads	−0.028	0.021		0.056	−0.005	0.018		0.010	−0.033	0.018		0.090
No bicycle infrastructure	—-	—-	—-	—-	—-	—-	—-	—-	—-	—-	—-	—-
W/ bicycle infrastructure	0.150	0.021	***	−0.304	0.101	0.018	***	−0.221	0.103	0.018	***	−0.285
Large roads	−0.035	0.018		0.070	−0.050	0.015	***	0.110	−0.060	0.015	***	0.167
No bicycle infrastructure	—-	—-	—-	—-	—-	—-	—-	—-	—-	—-	—-	—-
W/ painted bicycle lanes	−0.023	0.030		0.046	0.014	0.024		−0.030	−0.030	0.025		0.084
W/ protected bicycle lanes	0.175	0.018	***	−0.355	0.154	0.016	***	−0.338	0.122	0.015	***	−0.339
**Bicycle route classification**												
No classification	—-	—-	—-	—-	—-	—-	—-	—-	—-	—-	—-	—-
Cycle superhighway	0.042	0.016	**	−0.086	0.057	0.014	***	−0.126	0.040	0.014	**	−0.112
Proposed cycle superhighway	0.020	0.015		−0.041	0.055	0.012	***	−0.121	0.038	0.012	**	−0.106
**Land use, cycleways**												
High-rise urban areas	0.146	0.044	***	−0.296	0.119	0.041	**	−0.260	0.100	0.038	**	−0.277
Low-rise urban areas	—-	—-	—-	—-	—-	—-	—-	—-	—-	—-	—-	—-
Industrial areas	0.278	0.060	***	−0.564	0.221	0.052	***	−0.486	0.183	0.049	***	−0.509
Green areas	0.250	0.060	***	−0.506	0.243	0.054	***	−0.534	0.198	0.053	***	−0.549
Areas near water	0.105	0.057		−0.213	0.043	0.052		−0.093	−0.040	0.048		0.112
Open landscape	−0.131	0.103		0.266	−0.037	0.123		0.082	−0.045	0.092		0.125
**Land use, other infrastructure**												
High-rise urban areas	0.118	0.031	***	−0.239	0.088	0.024	***	−0.193	0.057	0.025	*	−0.157
Low-rise urban areas	—-	—-	—-	—-	—-	—-	—-	—-	—-	—-	—-	—-
Industrial areas	0.069	0.051		−0.141	0.072	0.038		−0.158	0.066	0.039		−0.184
Green areas	0.052	0.046		−0.106	0.054	0.037		−0.119	0.041	0.040		−0.113
Areas near water	0.162	0.039	***	−0.329	0.118	0.030	***	−0.259	0.091	0.031	**	−0.251
Open landscape	0.159	0.127		−0.323	0.083	0.096		−0.182	0.036	0.078		−0.100
**Elevation gain, $>$ 35 m/km**	−4.141	6.142		8.393	−7.559	3.682	*	16.595	−10.552	3.394	**	29.309
**Surface type**												
Asphalt	—-	—-	—-	—-	—-	—-	—-	—-	—-	—-	—-	—-
Cobblestones	−0.019	0.025		0.038	−0.014	0.019		0.030	−0.024	0.016		0.066
Gravel	−0.097	0.016	***	0.197	−0.086	0.015	***	0.189	−0.096	0.015	***	0.267
**Wrong way**	−0.253	0.010	***	0.512	−0.266	0.007	***	0.584	−0.251	0.007	***	0.698
N	917,525				1,866,767				2,972,994			

Table S5. The estimated parameters for the bicycle route choice model with 100, 200 and 400 Os and Ds. Standard errors are robust. The scaled values are scaled such that the parameter "Constant" has a value of 1. Significance levels are * *P*-value $\leq$ 0.001, ** *P*-value $\leq$ 0.01, and * *P*-value $\leq$ 0.05. *N* denotes the number of rows in the linear regression equation.**

Data	Data split A				Full data				Data split B			
Parameter	Coef.	Std. err.	*P*-val.	Scaled	Coef.	Std. err.	*P*-val.	Scaled	Coef.	Std. err.	*P*-val.	Scaled
**Constant**	−0.474	0.042	***	1	−0.456	0.027	***	1	−0.438	0.032	***	1
**Infrastructure**												
Stairs	−0.322	0.145	*	0.678	−0.342	0.096	***	0.750	−0.374	0.120	**	0.853
Pedestrian zones	−0.118	0.037	**	0.249	−0.121	0.025	***	0.265	−0.116	0.033	***	0.265
Footways	−0.090	0.018	***	0.191	−0.090	0.012	***	0.198	−0.090	0.017	***	0.205
Shared paths	−0.033	0.026		0.070	−0.026	0.016		0.057	−0.017	0.018		0.039
Living streets	−0.105	0.059		0.222	−0.051	0.039		0.113	−0.002	0.053		0.005
Cycleways	0.108	0.054	*	−0.229	0.089	0.037	*	−0.195	0.069	0.053		−0.158
Residential roads												
No bicycle infrastructure	——-	——-	——-	——-	——-	——-	——-	——-	——-	——-	——-	——-
W/ bicycle infrastructure	0.036	0.027		−0.077	0.065	0.019	***	−0.142	0.096	0.025	***	−0.219
Medium roads	0.010	0.022		−0.021	−0.005	0.018		0.010	−0.019	0.026		0.044
No bicycle infrastructure	——-	——-	——-	——-	——-	——-	——-	——-	——-	——-	——-	——-
W/ bicycle infrastructure	0.076	0.022	***	−0.159	0.101	0.018	***	−0.221	0.126	0.027	***	−0.287
Large roads	−0.052	0.021	*	0.109	−0.050	0.015	***	0.110	−0.049	0.020	*	0.112
No bicycle infrastructure	——-	——-	——-	——-	——-	——-	——-	——-	——-	——-	——-	——-
W/ painted bicycle lanes	0.003	0.033		−0.007	0.014	0.024		−0.030	0.020	0.034		−0.045
W/ protected bicycle lanes	0.161	0.023	***	−0.339	0.154	0.016	***	−0.338	0.145	0.022	***	−0.330
**Bicycle route classification**												
No classification	——-	——-	——-	——-	——-	——-	——-	——-	——-	——-	——-	——-
Cycle superhighway	0.056	0.019	**	−0.119	0.057	0.014	***	−0.126	0.059	0.020	**	−0.135
Proposed cycle superhighway	0.025	0.017		−0.053	0.055	0.012	***	−0.121	0.089	0.016	***	−0.202
**Land use, cycleways**												
High-rise urban areas	0.133	0.056	*	−0.280	0.119	0.041	**	−0.260	0.104	0.060		−0.238
Low-rise urban areas	——-	——-	——-	——-	——-	——-	——-	——-	——-	——-	——-	——-
Industrial areas	0.247	0.072	***	−0.520	0.221	0.052	***	−0.486	0.200	0.075	**	−0.458
Green areas	0.272	0.081	***	−0.574	0.243	0.054	***	−0.534	0.212	0.073	**	−0.484
Areas near water	0.083	0.071		−0.175	0.043	0.052		−0.093	0.001	0.075		−0.003
Open landscape	−0.011	0.144		0.023	−0.037	0.123		0.082	−0.081	0.212		0.184
**Land use, other infrastructure**												
High-rise urban areas	0.121	0.038	**	−0.256	0.088	0.024	***	−0.193	0.056	0.029		−0.128
Low-rise urban areas	——-	——-	——-	——-	——-	——-	——-	——-	——-	——-	——-	——-
Industrial areas	0.117	0.055	*	−0.246	0.072	0.038		−0.158	0.032	0.050		−0.072
Green areas	0.093	0.062		−0.197	0.054	0.037		−0.119	0.020	0.041		−0.045
Areas near water	0.136	0.048	**	−0.287	0.118	0.030	***	−0.259	0.102	0.037	**	−0.232
Open landscape	0.149	0.125		−0.314	0.083	0.096		−0.182	−0.018	0.125		0.040
**Elevation gain, $>$ 35 m/km**	−9.155	7.159		19.296	−7.559	3.682	*	16.595	−6.247	3.340		14.270
**Surface type**												
Asphalt	——-	——-	——-	——-	——-	——-	——-	——-	——-	——-	——-	——-
Cobblestones	−0.015	0.024		0.032	−0.014	0.019		0.030	−0.015	0.029		0.035
Gravel	−0.096	0.021	***	0.201	−0.086	0.015	***	0.189	−0.074	0.022	***	0.170
**Wrong way**	−0.256	0.010	***	0.539	−0.266	0.007	***	0.584	−0.276	0.010	***	0.631
N	929,569				1,866,767				937,198			

Table S6. The estimated parameters for the bicycle route choice model based on data split A, the full dataset, and data split B. Standard errors are robust. The scaled values are scaled such that the parameter "Constant" has a value of 1. Significance levels are * *P*-value $\leq$ 0.001, ** *P*-value $\leq$ 0.01, and * *P*-value $\leq$ 0.05. *N* denotes the number of rows in the linear regression equation.**

References

1. M Fosgerau, DL McFadden, A theory of the perturbed consumer with general budgets. *NBER Work. Pap.* pp. 1–27 (2012).
2. D Fudenberg, R Iijima, T Strzalecki, Stochastic Choice and Revealed Perturbed Utility. *Econometrica* **83**, 2371–2409 (2015).
3. R Allen, J Rehbeck, Identification With Additively Separable Heterogeneity. *Econometrica* **87**, 1021–1054 (2019).
4. R Allen, J Rehbeck, Revealed Stochastic Choice with Attributes. (2019).
5. D McFadden, Econometric Models of Probabilistic Choice in *Structural Analysis of Discrete Data with Econometric Applications*, eds. C Manski, D McFadden. (MIT Press, Cambridge, MA, USA), pp. 198–272 (1981).
6. M Fosgerau, M Paulsen, TK Rasmussen, A perturbed utility route choice model. *Transp. Res. Part C* **136** (2022).
7. G Menghini, N Carrasco, N Schüssler, KW Axhausen, Route choice of cyclists in zurich. *Transp. Res. Part A: Policy Pract.* **44**, 754–765 (2010).
8. J Hood, E Sall, B Charlton, A gps-based bicycle route choice model for san francisco, california. *Transp. letters* **3**, 63–75 (2011).
9. J Broach, J Dill, J Gliebe, Where do cyclists ride ? A route choice model developed with revealed preference GPS data. *Transp. Res. Part A: Policy Pract.* **46**, 1730–1740 (2012).
10. R Khatri, CR Cherry, SS Nambisan, LD Han, Modeling route choice of utilitarian bikeshare users with gps data. *Transp. Res. Rec.* **2587**, 141–149 (2016).
11. D Ton, O Cats, D Duives, S Hoogendoorn, How do people cycle in amsterdam, netherlands?: Estimating cyclists' route choice determinants with gps data from an urban areaa. *Transp. research record* **2662**, 75–82 (2017).
12. JM Casello, V Usyukov, Modeling cyclists' route choice based on gps data. *Transp. Res. Rec.* **2430**, 155–161 (2014).
13. P Chen, Q Shen, S Childress, A gps data-based analysis of built environment influences on bicyclist route preferences. *Int. J. Sustain. Transp.* **12**, 218–231 (2018).
14. M Ghanayim, S Bekhor, Modelling bicycle route choice using data from a GPS-assisted household survey. *Eur. J. Transp. Infrastructure Res.* **18**, 158–177 (2018).
15. CG Prato, K Halldórsdóttir, OA Nielsen, Evaluation of land-use and transport network effects on cyclists' route choices in the Copenhagen Region in value-of-distance space. *Int. J. Sustain. Transp.* **12**, 770–781 (2018).
16. A Sobhani, HA Aliabadi, B Farooq, Metropolis-hasting based expanded path size logit model for cyclists' route choice using gps data. *Int. J. Transp. Sci. Technol.* **8**, 161–175 (2019).
17. DT Fitch, SL Handy, Road environments and bicyclist route choice: The cases of davis and san francisco, ca. *J. Transp. Geogr.* **85**, 102705 (2020).
18. NR Shah, CR Cherry, Different safety awareness and route choice between frequent and infrequent bicyclists: Findings from revealed preference study using bikeshare data. *Transp. Res. Rec.* **2675**, 269–279 (2021).
19. SH Cho, D Shin, Estimation of route choice behaviors of bike-sharing users as first-and last-mile trips for introduction of mobility-as-a-service (maas). *KSCE J. Civ. Eng.* pp. 1–12 (2022).
20. E Frejinger, M Bierlaire, M Ben-Akiva, Sampling of alternatives for route choice modeling. *Transp. Res. Part B: Methodol.* **43**, 984–994 (2009).
21. T Koch, E Dugundji, Limitations of recursive logit for inverse reinforcement learning of bicycle route choice behavior in amsterdam. *Procedia Comput. Sci.* **184**, 492–499 (2021).
22. M Fosgerau, E Frejinger, A Karlstrom, A link based network route choice model with unrestricted choice set. *Transp. Res. Part B: Methodol.* **56**, 70–80 (2013).
23. T Mai, M Fosgerau, E Frejinger, A nested recursive logit model for route choice analysis. *Transp. Res. Part B: Methodol.* **75**, 100–112 (2015).
24. M Zimmermann, T Mai, E Frejinger, Bike route choice modeling using GPS data without choice sets of paths. *Transp. Res. Part C: Emerg. Technol.* **75**, 183–196 (2017).
25. J Pucher, J Dill, S Handy, Infrastructure, programs, and policies to increase bicycling: An international review. *Prev. Medicine* **50**, S106–S125 (2010).
26. R Buehler, J Dill, Bikeway networks: A review of effects on cycling. *Transp. Rev.* **36**, 9–27 (2016).
27. R Aldred, Built Environment Interventions to Increase Active Travel: a Critical Review and Discussion. *Curr. Environ. Heal. Reports* **6**, 309–315 (2019).
28. K van Goeverden, TS Nielsen, H Harder, R van Nes, Interventions in Bicycle Infrastructure, Lessons from Dutch and Danish Cases. *Transp. Res. Procedia* **10**, 403–412 (2015).
29. FJM Mölenberg, J Panter, A Burdorf, FJ van Lenthe, A systematic review of the effect of infrastructural interventions to promote cycling: strengthening causal inference from observational data. *Int. J. Behav. Nutr. Phys. Activity* **16**, 93 (2019).
30. D Merom, A Bauman, P Vita, G Close, An environmental intervention to promote walking and cycling—the impact of a newly constructed Rail Trail in Western Sydney. *Prev. Medicine* **36**, 235–242 (2003).
31. KR Evenson, AH Herring, SL Huston, Evaluating change in physical activity with the building of a multi-use trail. *Am. J. Prev. Medicine* **28**, 177–185 (2005).
32. KJ Krizek, G Barnes, K Thompson, Analyzing the Effect of Bicycle Facilities on Commute Mode Share over Time. *J. Urban Plan. Dev.* **135**, 66–73 (2009).
33. A Goodman, S Sahlqvist, D Ogilvie, New Walking and Cycling Routes and Increased Physical Activity: One- and 2-Year Findings From the UK iConnect Study. *Am. J. Public Heal.* **104**, e38–e46 (2014).

34. H Skov-Petersen, JB Jacobsen, SE Vedel, SNT Alexander, S Rask, Effects of upgrading to cycle highways-an analysis of demand induction, use patterns and satisfaction before and after. *J. transport geography* **64**, 203–210 (2017).
35. J Hong, DP McArthur, M Livingston, The evaluation of large cycling infrastructure investments in glasgow using crowdsourced cycle data. *Transportation* **47**, 2859–2872 (2020).
36. AC Nelson, D Allen, If You Build Them, Commuters Will Use Them: Association Between Bicycle Facilities and Bicycle Commuting. *Transp. Res. Rec. J. Transp. Res. Board* **1578**, 79–83 (1997).
37. J Dill, T Carr, Bicycle Commuting and Facilities in Major U.S. Cities: If You Build Them, Commuters Will Use Them. *Transp. Res. Rec.* pp. 116–123 (2003).
38. J Pucher, R Buehler, Cycling trends & policies in Canadian cities. *World Transp. Policy & Pract.* **11**, 43–61 (2005).
39. J Parkin, M Wardman, M Page, Estimation of the determinants of bicycle mode share for the journey to work using census data. *Transportation* **35**, 93–109 (2008).
40. JE Schoner, DM Levinson, The missing link: bicycle infrastructure networks and ridership in 74 us cities. *Transportation* **41**, 1187–1204 (2014).
41. A Osama, T Sayed, AY Bigazzi, Models for estimating zone-level bike kilometers traveled using bike network, land use, and road facility variables. *Transp. Res. Part A: Policy Pract.* **96**, 14–28 (2017).
42. TAS Nielsen, H Skov-Petersen, Bikeability – Urban structures supporting cycling. Effects of local, urban and regional scale urban form factors on cycling from home and workplace locations in Denmark. *J. Transp. Geogr.* **69**, 36–44 (2018).
43. S Ryan, A Garate, D Foote, D Foote, A Micro-Scale Analysis of Cycling Demand, Safety, and Network Quality. *Mineta Transp. Inst. Publ.* (2020).
44. G Vandenbulcke, et al., Cycle commuting in Belgium: Spatial determinants and 're-cycling' strategies. *Transp. Res. Part A: Policy Pract.* **45**, 118–137 (2011).
45. DJ Fagnant, K Kockelman, A direct-demand model for bicycle counts: the impacts of level of service and other factors. *Environ. Plan. B: Plan. Des.* **43**, 93–107 (2016).
46. S Kraus, N Koch, Provisional COVID-19 infrastructure induces large, rapid increases in cycling. *Proc. Natl. Acad. Sci. United States Am.* **118** (2021).
47. M Iacono, K Krizek, A El-Geneidy, Access to destinations: How close is close enough? estimating accurate distance decay functions for multiple modes and different purposes, (Hubert H. Humphrey Institute of Public Affairs, University of Minnesota), Technical report (2008).
48. R Lovelace, et al., The propensity to cycle tool: An open source online system for sustainable transport planning. *J. Transp. Land Use* **10**, 505–528 (2017).
49. C Liu, A Tapani, I Kristoffersson, C Rydergren, D Jonsson, Development of a large-scale transport model with focus on cycling. *Transp. Res. Part A: Policy Pract.* **134**, 164–183 (2020).
50. M Hallberg, TK Rasmussen, J Rich, Modelling the impact of cycle superhighways and electric bicycles. *Transp. Res. Part A: Policy Pract.* **149**, 397–418 (2021).
51. J Rich, AF Jensen, N Pilegaard, M Hallberg, Cost-benefit of bicycle infrastructure with e-bikes and cycle superhighways. *Case Stud. on Transp. Policy* **9**, 608–615 (2021).
52. A Agarwal, D Ziemke, K Nagel, Bicycle superhighway: An environmentally sustainable policy for urban transport. *Transp. Res. Part A: Policy Pract.* **137**, 519–540 (2020).
53. OpenStreetMap contributors, Dump retrieved from https://overpass-api.de (https://www.openstreetmap.org) (2022).
54. FOT-Kort10, FOT-kort10 (https://download.kortforsyningen.dk/) (2018).
55. Hövding ApS, Cyclist trajectory data (https://www.hovding.com/) (2021).
56. Technical University of Denmark, Danish National Travel Survey (2006–2021).
57. JH Haunert, B Budig, An algorithm for map matching given incomplete road data. *GIS: Proc. ACM Int. Symp. on Adv. Geogr. Inf. Syst.* pp. 510–513 (2012).
58. D Ton, D Duives, O Cats, S Hoogendoorn, Evaluating a data-driven approach for choice set identification using GPS bicycle route choice data from Amsterdam. *Travel. Behav. Soc.* **13**, 105–117 (2018).
59. T Koch, L Knapen, E Dugundji, Path complexity and bicyclist route choice set quality assessment. *Pers. Ubiquitous Comput.* **25**, 63–75 (2021).
60. MOSEK ApS, *MOSEK Fusion API for Python 9.3.20*, (2019).
61. E Heinen, B van Wee, K Maat, Commuting by Bicycle: An Overview of the Literature. *Transp. Rev.* **30**, 59–96 (2010).
62. G Liu, M te Brömmelstroet, S Krishnamurthy, P van Wesemael, Practitioners' perspective on user experience and design of cycle highways. *Transp. Res. Interdiscip. Perspectives* **1**, 100010 (2019).
63. Region Hovedstaden, Cykelregnskab 2020, (AFRY for Region Hovedstaden), Technical report (2020).
64. M Fosgerau, N Pilegaard, The Rule-of-a-Half and Interpreting the Consumer Surplus as Accessibility in *International Encyclopedia of Transportation*, ed. R Vickerman. (Elsevier), pp. 237–241 (2021).
65. Technical University of Denmark, Transport Economic Unit Prices vers. 2.0 (2022).
66. EA Nadaraya, On Estimating Regression. *Theory Probab. & Its Appl.* **9**, 141–142 (1964).
67. GS Watson, Smooth Regression Analysis. *Sankhya: The Indian J. Stat. Ser. A* **26**, 175–184 (1964).
